# Northeast Yucatan hurricane activity during the Maya Classic and Postclassic periods

**DOI:** 10.1038/s41598-022-22756-2

**Published:** 2022-11-22

**Authors:** Richard M. Sullivan, Peter J. van Hengstum, Jeffrey P. Donnelly, Anne E. Tamalavage, Tyler S. Winkler, Shawna N. Little, Luis Mejia-Ortiz, Eduard G. Reinhardt, Sam Meacham, Courtney Schumacher, Robert Korty

**Affiliations:** 1 Department of Oceanography, Texas A&M University, College Station, TX 77843 US; 2 Department of Marine and Coastal Environmental Science, Texas A&M University at Galveston, 1001 Texas Clipper Road, Galveston, TX 77553 US; 3 Geology & Geophysics, Woods Hole Oceanographic Institution, 266 Woods Hole Rd, Woods Hole, MA 02543 US; 4 Département de Sciences Biologiques, Université de Montréal, 1375 Avenue Thérèse-Lavoie-Roux, Montréal, QC H2V 0B3 Canada; 5 Department of Marine Biology, Texas A&M University at Galveston, 1001 Texas Clipper Road, Galveston, TX 77553 US; 6 División de Desarrollo Sustentable, Universidad Autónoma del Estado de Quintana Roo, Av. Andrés Quintana Roo s/n, 77600 Cozumel, Quintana Roo Mexico; 7 School of Geography and Earth Sciences, McMaster University, Hamilton, ON L8S 4K1 Canada; 8 CINDAQ - El Centro Investigador del Sistema Acuífero de Quintana Roo, A.C., Puerto Aventuras, Quintana Roo Mexico; 9 Department of Atmospheric Sciences, Texas A&M University, College Station, TX 77843 US

**Keywords:** Palaeoclimate, Natural hazards

## Abstract

The collapse of the Maya civilization in the late 1st/early 2nd millennium CE has been attributed to multiple internal and external causes including overpopulation, increased warfare, and environmental deterioration. Yet the role hurricanes may have played in the fracturing of Maya socio-political networks, site abandonment, and cultural reconfiguration remains unexplored. Here we present a 2200 yearlong hurricane record developed from sediment recovered from a flooded cenote on the northeastern Yucatan peninsula. The sediment archive contains fine grain autogenic carbonate interspersed with anomalous deposits of coarse carbonate material that we interpret as evidence of local hurricane activity. This interpretation is supported by the correlation between the multi-decadal distribution of recent coarse beds and the temporal distribution of modern regional landfalling storms. In total, this record allows us to reconstruct the variable hurricane conditions impacting the northern lowland Maya during the Late Preclassic, Classic, and Postclassic Periods. Strikingly, persistent above-average hurricane frequency between ~ 700 and 1450 CE encompasses the Maya Terminal Classic Phase, the declines of Chichén Itza, Cobá, and subsequent rise and fall of the Mayapán Confederacy. This suggests that hurricanes may have posed an additional environmental stressor necessary of consideration when examining the Postclassic transformation of northern Maya polities.

## Introduction

Memorialized today by their monumental architecture, the Classic Maya (300 to ~ 900 CE) represented one of the largest and most socially complex indigenous cultures of the Americas. It is for this reason that their abrupt cultural disintegration and realignment in the late 1st millennium has encouraged continuous academic scrutiny and speculation. Numerous socially-linked factors, including overpopulation^1^, deforestation^2–4^, and inter-polity warfare^5–7^ have been proposed and examined as contributing to the dissolution of the Maya socio-political networks. Additionally, regional paleoclimate reconstructions consistently reveal recurrent and prolonged episodes of drought during the Terminal Classic Phase (TCP, ~ 800 to ~ 1050 CE). While the precise contribution of aridity to the Maya collapse is unknown, droughts may cause social fragmentation and encourage human migration^8–10^. The combined impact of these multiple stressors would have further frayed the Maya social fabric, ultimately leading to regional instability^6,11–16^. Indeed, archaeological evidence suggests that the Maya Classic decline was asynchronous across the Yucatan with northern cities flourishing as southern polities were abandoned^1,5,17,18^. This spatial and chronological distribution of political fracturing and demographic change indicates that the Maya experienced a combination of allochronic social and natural stressors that ultimately led to a population decline of nearly 90% over this period^19^.

**Figure 1:**
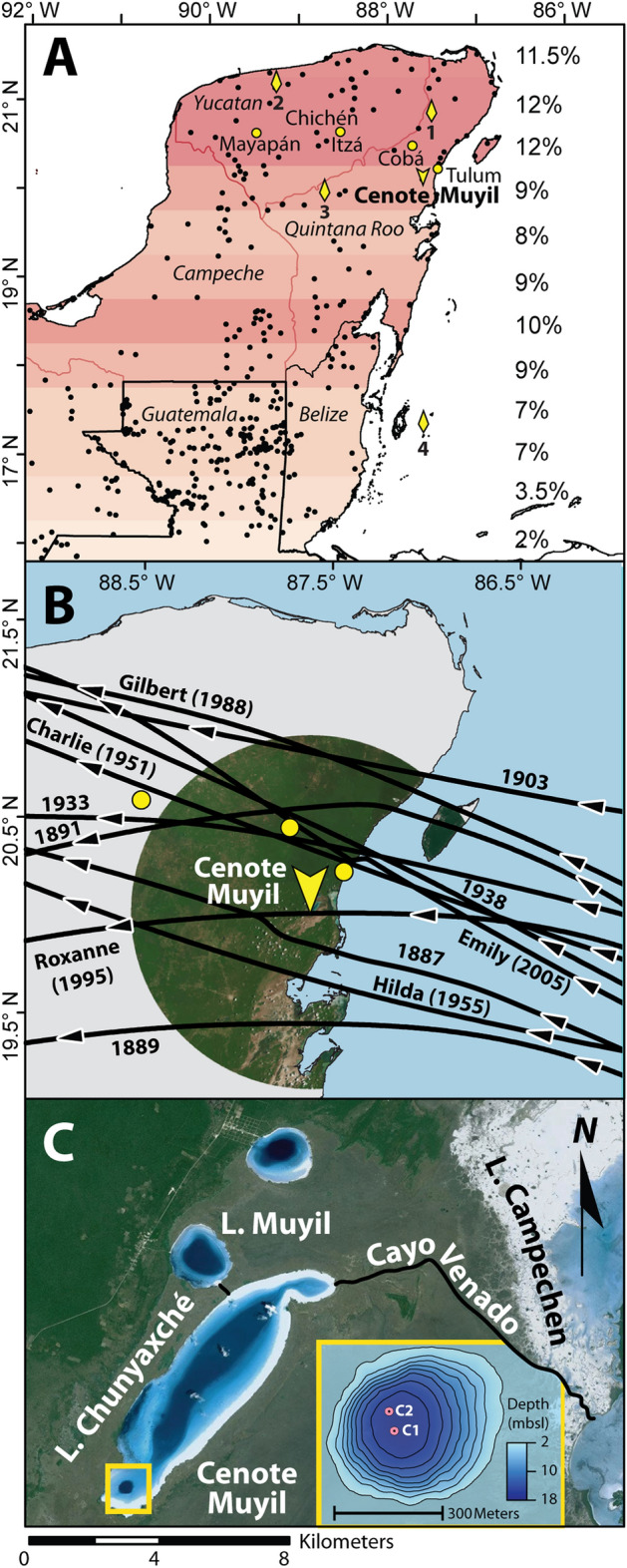
(**A**) Distribution of Maya settlements and locations discussed in text. Cenote Muyil is marked by a chevron. Paleoclimate studies discussed in this manuscript are shown as yellow diamonds (1. Punta Laguna, 2. Chaac, 3. Laguna Chichancanab, and 4. Great Blue Hole Belize). Colored bars depict the percentage of tropical storm events, or greater, (binned at 0.5° latitude) from NOAA’s Best Track Dataset that intersected with the Yucatan Peninsula between 1920 and 2020 CE. Country borders are outlined in black and Mexico state borders in red. Black dots mark locations of known Maya sites. Yellow dots denote Tulum, Cobá, Chichén Itza, and Mayapán. (**B**) All storms Cat 3 and above that have passed within 100 km of Cenote Muyil (chevron) since 1850 CE. Yellow dots mark (east to west) Tulum, Cobá, and Chichén Itza. (**C**) Aerial view of research area. The Cayo Venado Canal is shown in black. Inset shows core locations and Muyil bathymetry.

In addition to drought, hurricane strikes have also been a catalyst for abrupt population and settlement changes and pose a continuing threat to human life, especially on low-lying landscapes like the Yucatan Peninsula. Between 1920 and 2020 CE the Yucatan was struck by 112 tropical storms, including 18 major hurricane events (≥ Category 3 on the Saffir-Simpson Scale), 72% of which made landfall above 18.25° N (a region coterminous with the northern Maya Lowlands) (Fig. 1). Prior work^20^ has demonstrated that modern precipitation variability over the Yucatan can positively covary with the frequency of tropical cyclones passing within 100 km of the peninsula. Though this suggests that paleo-rainfall reconstructions may reflect regional hurricane activity^20^, recent work has shown that multi-decadal shifts between intervals of more (less) positive water balance need not correlate with increased (decreased) hurricane activity on paleo time scales^21^. Furthermore, analysis shows that modern multidecadal precipitation patterns over Muyil do not positively correlate with modern multi-decadal patterns of major hurricane occurrence (Figure S1). Considering that many paleo-proxy reconstructions are time-averaged and provide variable temporal resolutions, we should not expect paleo-rainfall records to accurately depict past hurricane activity.

While drought and hurricanes may occupy hydrological antipodes, the occurrence of one does not preclude the occurrence of the other. For example, deforestation may result in a reduction of local rainfall^2,3^ decoupled from the synoptic scale hydroclimate mechanisms that modulate hurricane behavior^22,23^. Local aridity may impede storm intensification through dry air intrusion, but storms with a sufficient moisture budget may maintain their strength or continue to intensify prior to landfall^24^. Considering this relationship, it follows that existing moisture balance reconstructions alone provide incomplete insight into past Yucatan hydroclimate instability. Independent reconstructions focused solely on hurricane activity are needed to assess the synchroneity between the frequency of extreme storm events and the cultural realignment of the ancient Maya. However, no such reconstructions exist to further our understanding of this volatile period.

Here we present a ~ 2200 year record of hurricane activity extracted from Cenote Muyil in Laguna Chunyaxché situated on the northeastern Yucatan Peninsula (Fig. 1**)**. This work encompasses the entirety of the Maya Classic and Postclassic Periods and represents the first hurricane reconstruction from the northern Lowlands. Our results show a distinct and persistent increase in hurricane frequency beginning ~ 700 CE, which may have further impacted a civilization already contending with social conflicts and environmental degradation.

## Study site

Laguna Chunyaxché (20.04° N, 87.06° W) is a groundwater-fed 20 km^2^ freshwater lagoon located ~ 30 km south of Tulum (Fig. 1) and is administered as part of the Sian Ka’an bioreserve, a UNESCO world heritage site. The lagoon has a maximum measured depth of 25 m and is just over 8 km from the coast at its most proximal point. Chunyaxché is connected to Laguna Muyil to the west and Laguna Campechen on the east by the Cayo Venado canal, which was utilized by the native Maya during maritime trade^25^. The lagoon network is separated from the Caribbean Sea by a ~ 14 km long spit that protects the mangroves (e.g. *Rhizophora mangle* and *Conocarpus erectus)*, herbaceous dominated wetlands^26^, and local Maya architecture from the open ocean and storm-driven marine overwash. Despite this protection, the region remains sensitive to storm impacts, which have previously lead to substantial flooding and wind damage resulting in observed losses of up to 83% of vegetative cover^27,28^. Over 50 tropical cyclones (of tropical storm strength or greater, winds ≥ 34 kts) have passed within 100 km of Chunyaxché since 1858 CE, with 11 making landfall as major hurricanes (Fig. 1B). The 7 km of available fetch along Chunyaxché’s NE/SW axis means that near-passing storms may generate significant wave heights within the lagoon. Northeasterly winds comparable to a Category 3 hurricane (sustained winds > 96 kt) may produce wave heights exceeding 3.6 m. Wave heights within Chunyaxché may surpass 6.0 m when exposed to sustained Category 5 wind speeds such as those generated by Hurricane Gilbert in 1988 (> 137 kts).

At the southwest end of Chunyaxché, and ~ 500 m from the fringing mangroves, is a 17 m deep sinkhole referred to in this study as Cenote Muyil, so named for the adjacent Maya archaeological site. Bathymetric mapping of the cenote revealed a simple bowl-like sedimentary basin, with the deepest area located roughly in the middle (Fig. 1, S2). Basin morphology does not suggest underwater limestone ledges or caves, though firsthand observations (i.e., SCUBA) are unavailable. Previous work exploring the water chemistry of the Sian Ka’an bioreserve determined the local ground water to be largely fresh, calcite-saturated, and phosphorus-limited while containing little to no sulphate^29^. Cenote Muyil itself contains an oxygenated (5.5 to 7.75 mg l^-1^) oligohaline (0.69 psu) water column with an alkaline pH between 8.1 and 8.3 (Figure S3). While direct measurements were not taken, the system is most likely saturated with respect to carbonate since groundwater is derived from the local limestone aquifer and the cenote does not receive river discharge.

The ancient Maya settlement at Muyil is situated ~ 14 km inland of the modern Caribbean coast and connects to the ocean via the network of canals and lagoons. Through much of its history Muyil likely served as a port and coastal trading hub that facilitated exchange between the inland metropolitan centers in the north and the more southern regions of Maya influence^25^. Occupation at Muyil began around 300 BCE^25^ during the Late Preclassic Period (300 BCE to ~ 300 CE) and likely functioned as an active trading center with northern polities throughout the Early (300 CE to 600 CE) and Late Classic (600 CE to 900 CE) Periods. The site remained continuously occupied into the late Postclassic (1200 CE to 1500 CE), which possibly indicates increased reliance on coastal supply and trade routes as inland populations decreased^18,30,31^. Abandonment of Muyil was contemporaneous with European conquest in the early sixteenth century^25^.

## Cenote Muyil sediment record

### Stratigraphy

**Figure 2:**
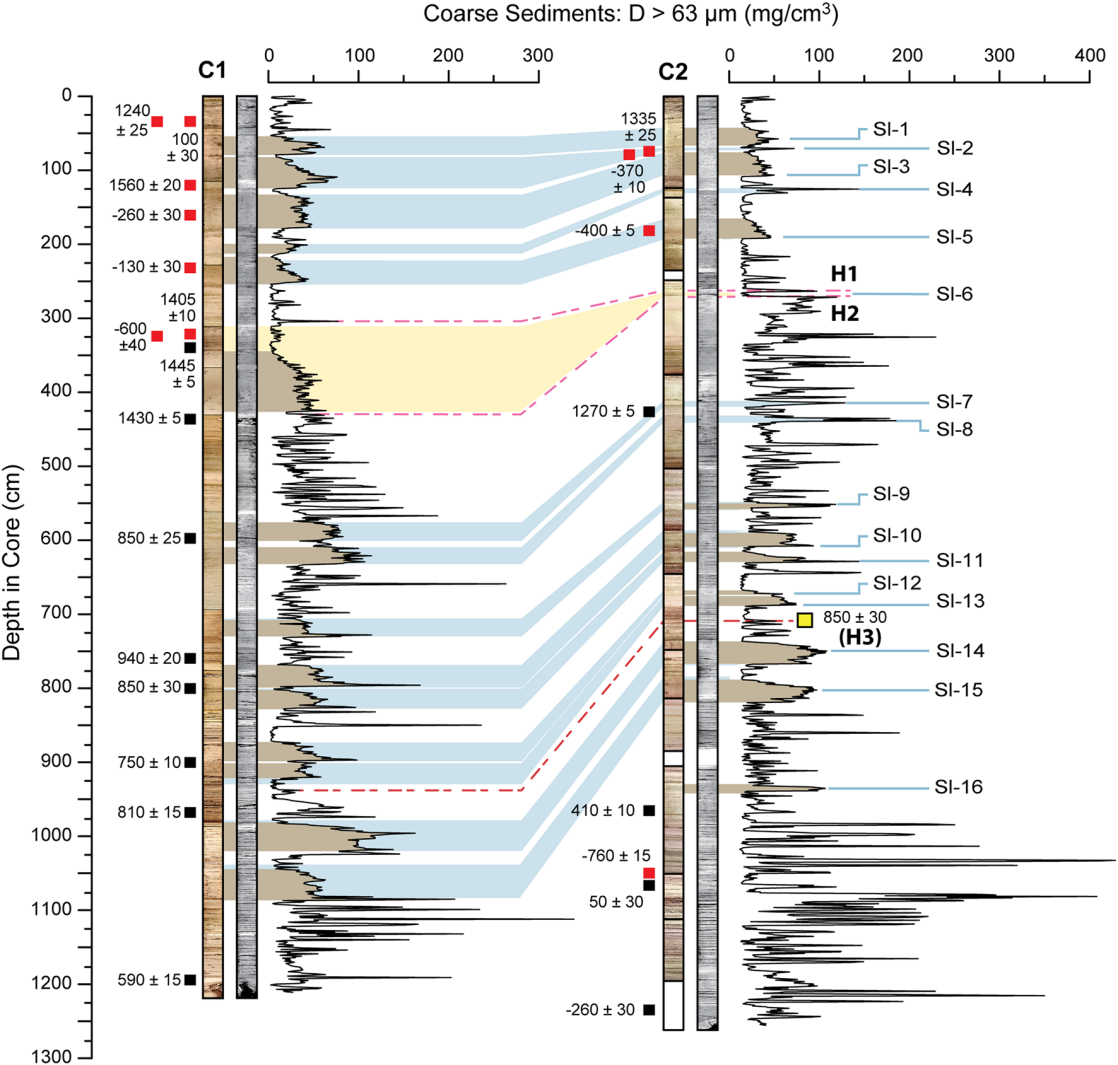
Optical and radiograph images, along with downcore coarse particle content for C1 and C2 from Cenote Muyil. Position of radiocarbon dates and associated calibrated Common Era ages (negative sign denotes BCE) are listed to the left of each core. Red squares denote age results that were excluded from the final Bayesian age model. Blue shading corresponds to slump deposits between the two cores. Slumps numbered on the right. Stratigraphic tie points H1 and H2 bracket the hiatus in C2. The yellow region indicates material present in C1 that is missing in C2, including the Sl-6 slump feature. H3 marks the location of the AMS age from C2 incorporated into the Bayesian age model of C1.

In 2017, a submersible vibracoring survey recovered two continuous (> 12 m long) sediment cores from the central region of Cenote Muyil (*see Methods in Supplemental Text S1*). The sedimentary infill consists of fine-grained lacustrine carbonate marl interspersed with amorphous coarse-grained (> 63 μm diameter) carbonate sand particles (Figs. 2, S4), occasional freshwater snail shell material (e.g., *Hydrobia*, *Pyrgophorus*), and limited quantities of calcite tubules and freshwater ostracod valves (e.g., *Cytheridella ilosvayi*). Photographs and x-radiographs from both cores document intervals with distinct horizontal laminations and bedding (Figs. 2, S4), which indicates minimal vertical sediment mixing from either bioturbation or physical reworking. No marine sedimentary particles (e.g., foraminifera), layers of shell hash (freshwater or marine), or layers of gypsum were observed in the stratigraphy. This suggests that conditions in the overlying water column remained broadly consistent throughout the Common Era and received no direct sedimentary input from the adjacent marine environment through coastal flooding. Despite the proximity of the heavily vegetated shoreline (~ 500 m), the cores contained few particles that could be confidently sourced to the terrestrial landscape or wetlands (e.g., mangrove leaves, twigs). In tropical North Atlantic karstic lakes lacustrine marl deposition occurs when carbonate-saturated groundwater decalcifies from evaporation, CO_2_ degases from the mixing of thermally distinct water bodies, or from bicarbonate assimilation from algae or macrophytes^32,33^. It is likely that similar physical and biologic processes promote carbonate marl deposition in Muyil. The two most noticeable attributes of the coarse particle distribution are i) a tripartite textural division that becomes finer towards the surface (Figure S5), and ii) that thin (~ 1 to 2 cm) coarse beds are occasionally interrupted by much thicker (> 5 cm) and distinct coarse slump-like features (Figs. 2, S6, S7). Nearly all these thicker deposits consist of the same carbonate mud and shell material found in the narrower coarse beds, lack any internal stratigraphic structure, and many exhibit a fining upward sequence. This suggests that these features, all or in part, may represent instantaneous slump deposits potentially driven by gravitationally forced sediment flow. Fifteen slump features were identified in C1 and 11 were identified within C2. Ten of the slump features identified in C1 also presented as slumps in C2. Four of the remaining features in C1 appeared as narrow (< 5 cm width) coarse beds in C2. Finally, the largest slump in C1 (width 85 cm, referred to as Sl-6) matches stratigraphically to a hiatus in C2 (marked by yellow in Figs. 2, S6). It is probable that the event that triggered the Sl-6 slump in C1 was responsible for the hiatus in C2. For further discussion of the slumps, see *Supplemental Text S2*.

Despite being similar in length and recovered from identical depths (17 mbsl), the two cores represent different spans of time. Core 1 has a basal age of 602 CE and encompasses 1415 years, while C2 has a basal age of 289 BCE and encompasses 2199 years (excluding the 105-year interval lost in the hiatus). This difference in basal ages is unsurprising considering the hiatus in C2 and thicker slump deposits within C1 that are not present in C2.

The age-depth models for these cores were developed from plant fragments (e.g., seeds and leaves, *n* = *13*) that were radiocarbon dated at the National Ocean Sciences Accelerator Mass Spectrometry (NOSAMS) facility at the Woods Hole Oceanographic Institution (Figure S8). Twenty-four radiocarbon dates were initially extracted from the two cores despite a dearth of identifiable terrestrial organic remains within the sediment (Fig. 2). Of those 24 dates, 11 were excluded from the age model since they did not depict a coherent age-depth relationship consistent with their stratigraphic order. Ten of the 11 excluded dates were located above the Sl-6 slump feature. These dates may have been older reworked remains or potentially obtained from aquatic plant materials that were uniformly impacted by hard-water effects, and thus the ages skewed older than was reasonable for the surrounding stratigraphic unit (*See Supplemental Text S4*). The lack of stratigraphically consistent ages in the upper sediment presented a significant challenge in deciphering the more recent portions of the Muyil archive and introduced a degree of uncertainty that cannot be fully addressed. However, material extracted from C1 immediately above and below the Sl-6 slump constrained the timing of this event to the first half of the fifteenth century. All radiocarbon ages below this feature were included in the final age models for both cores, apart from one anomalously old date towards the base of C2 (*for further discussion see Supplemental Text S3*). This means that while we may be unable to adequately resolve the timing of individual depositional events occurring in the last 500 years, we are confident in the timing of deposits that predate ~ 1450 CE.

The tripartite lithostratigraphic divisions (*Supplemental Text S3*) provide a first-order test of the validity of the radiocarbon results between the two cores. The oldest phase (Phase 3) occurs from the base of C2 to 984 cm (290 BCE to 325 CE), which is the interval with the coarsest event bed texture (181.3 ± 98 mg/cm^3^) (Figure S5, Table S1). Since the base of C1 is considerably younger, it is consistent that Phase 3 is not depicted in C1. Phase 2 is present in both C1 (1212 cm to 425 cm; 600 CE to 1445 CE) and C2 (984 cm to 270 cm; 330 CE to 1445 CE) and displays similarly textured event beds of 101 ± 63.8 mg/cm^3^ and 98.3 ± 43.7 mg/cm^3^, respectively. Finally, there is an abrupt decrease in coarse particle deposition in C1 above the Sl-6 feature at 340 cm core depth. This phase spans from 1445 CE to present and has a mean event bed density of 42.7 ± 18.5 mg/cm^3^. A similar shift (42.2 ± 29.6 mg/cm^3^) occurs above the hiatus in C2 at a depth of 270 cm, which coincides with a date of ~ 1550 CE. The phases also display distinct sedimentation rates when the intervals inferred to be slump deposits are considered instantaneous depositional events. In C1 the sedimentation rate decreased from 0.73 cm/year in Phase 2 to 0.31 cm/year in the Phase 1. In C2 sediment accumulated at a rate of 0.41 cm/year in Phase 1, 0.59 cm/year in Phase 2, and 0.45 cm/year in Phase 3. While these changes are modest, they are apparent and indicate an increased rate of sediment deposition from ~ 330 CE to ~ 1450 CE. The stratigraphy also preserves numerous narrow (~ 1 to 2 cm) coarse particle horizons within these depositional phases. The majority of these narrow beds are found in the first half of Phase 3 (< 100 CE) and in the later portion of Phase 2 (> 700 CE). Phase 1 contains the least number of coarse beds.

### Storms and tempestites

On carbonate platforms, intense hurricane events and their associated wave action can re-suspend and re-deposit existing sedimentary reserves in both narrow-diameter lake settings^34^ and large banktop marine lagoons^35^. Following storm passage, attenuation of local hydrodynamic flow causes coarse particles to settle-out of suspension which can then be preserved in the sedimentary architecture of adjoining flooded sinkholes and blue holes^34,35^. Similar processes are likely responsible for coarse particle deposition within Cenote Muyil. Hurricane force winds oriented along Chunyaxché’s NE/SW axis may generate internal waves capable of transporting coarse sedimentary particles into the cenote, which are archived as anomalous course deposits that are juxtaposed against the fine-grained autogenic carbonate sedimentary matrix. The orientation of the lagoon and its elongated shape suggest that Cenote Muyil may be most sensitive to storms passing to the south as this provides the greatest fetch for the storm’s stronger northeasterly winds.

**Figure 3:**
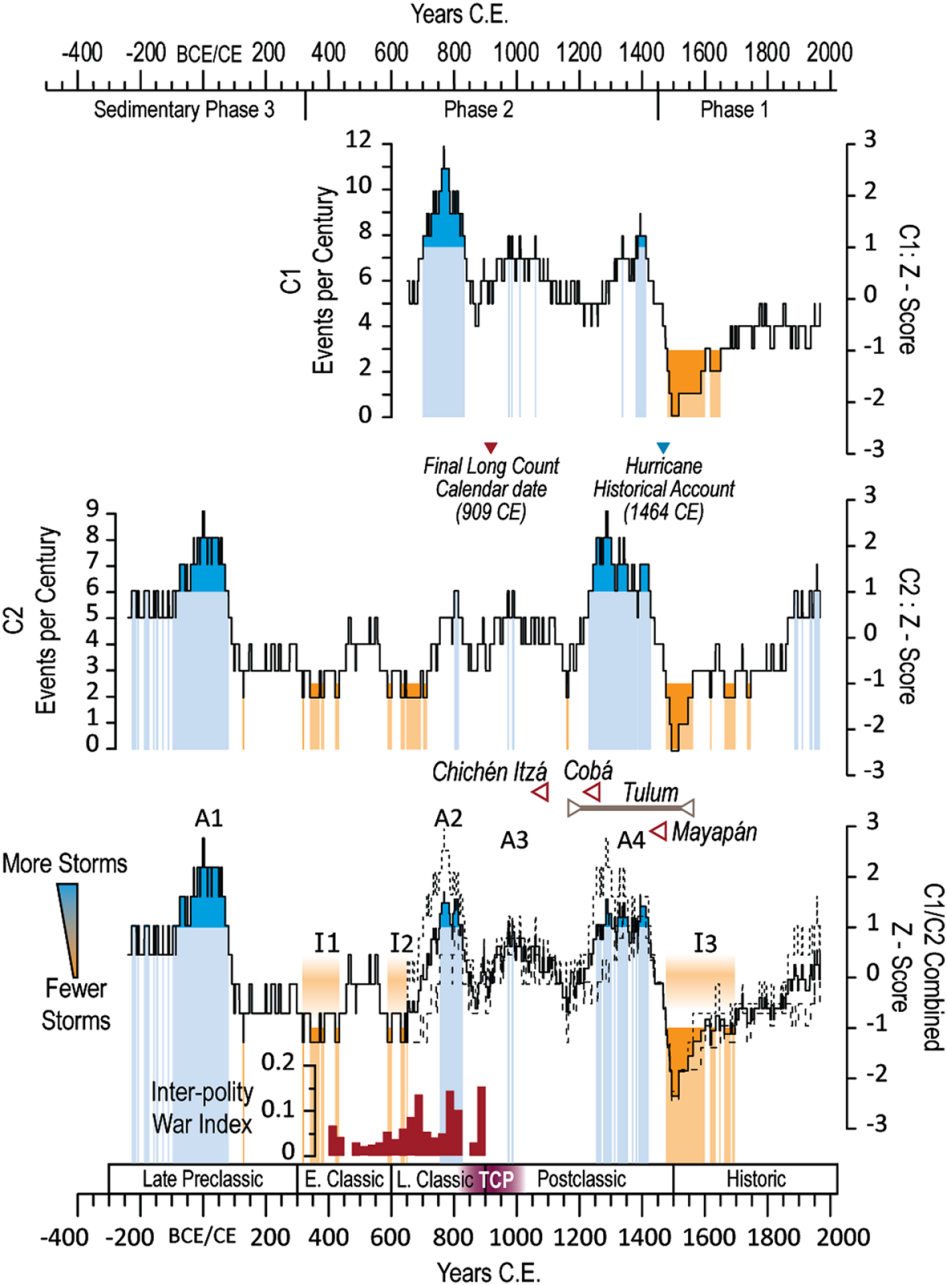
Z-Scores and depositional event counts per 100 years (century) for Muyil C1 (top) and C2 (middle). Blue (orange) shaded regions are intervals where event frequency exceeds (falls below) 1σ, referred to in the main text as *Active* (A1 to A4) and *Inactive* (I1 to I3) intervals. The lower plot represents the combined average of the C1 and C2 Z-Scores. Dashed lines mark the maximum and minimum bounds spanning the interval where the core sedimentary records overlap in time. The timing of the three sedimentary phases observed in Cenote Muyil is delineated along the top year axis. Maya cultural phases, including the Terminal Classic Phase (TCP) are listed above the lower year axis. Final known Long Count Calendar date is shown at 909 CE ^11^ and the recorded hurricane of 1464 CE is shown as the blue triangle ^62^. The declines of Chichén Itza, Cobá, and Mayapán are indicated in red and the occupation period of the coastal settlement of Tulum is shown in grey. The Inter-polity war index is adapted from Kennett et al. ^6^, which shows increased war activities during active interval A2.

Both cores from Muyil contain sediment beds that satisfy the criteria for anomalous coarse grain deposition (*see supplementary Methods S1.2.*) associated with the occurrence of high energy events. C1 contains a total of 74 depositional event beds while C2 contains 99 (59 within the 1415 years of temporal overlap between the cores) (Figures S9). The frequency of anomalous coarse bed deposition varies significantly for the duration of each record. We can apply a 100-year moving window (excluding the 50-year end points on either side) to highlight centennial variability in anomalous coarse deposition (Fig. 3). The mean and 1σ event frequency as calculated by the 100-year moving count can be used to identify intervals of anomalously high or low event occurrence. This method yields an average frequency (± 1σ) of 5.21 ± 2.30 events per century for C1 and 4.22 ± 1.38 events per century for C2. This gives us a 1σ thresholds in C1 of 3.44 to 8.11 events per century and 2.50 to 5.95 events per century in C2. Frequency counts occurring above (below) these limits are considered anomalously active (inactive) periods. For the purposes of discussion, successive active (inactive) intervals occurring within 50 years of each other can be grouped into clusters. The combined C1 and C2 frequency record, presented as the mean Z-score (Fig. 3), contains four active clusters (A1: 215 BCE to 80 CE, A2: 755 CE to 825 CE, A3: 975 to 985 CE, and A4: 1285 to 1420 CE) and three inactive clusters (I1: 315 to 435 CE, I2: 590 to 650 CE, I3: 1495 to 1745 CE). It should be noted that these counts assume the slump-like deposits within the cenote are evidence of hurricane strikes. While slumps may be triggered by events unrelated to tropical cyclone activity *(Supplemental Text S2)* there is currently no unbiased method for determining whether an individual slump was caused by a slow-moving hurricane or some other mechanism. Furthermore, excluding all slump features from the event count assumes that these features can confidently be attributed to depositional processes other than hurricanes. Regardless, since the number of these slumps is so few relative to the narrower coarse deposits, the overall pattern of centennial scale tempestite variability within Muyil does not appreciably change whether slumps are included or excluded from the final event count (Figure S10).

### Storm record

We infer that anomalous coarse beds archived within Cenote Muyil indicate an elevated hydrodynamical flow and wave climate induced by high energy events, such as near-to-direct passing hurricanes. According to the observational record, eight major events (≥ Category 3 on the Saffir-Simpson scale) have passed within 100 km of Cenote Muyil during the twentieth and twenty-first centuries. Cores C1 and C2 contain six and nine coarse deposits respectively since 1900 CE and include a notable absence of coarse beds during the 1960s and 1970s when no major hurricanes hit the northern Yucatan Peninsula. The comparable quantity of recent coarse deposits to historically recorded storms in the region lends credence to the interpretation that coarse beds in Cenote Muyil reflect local major hurricane activity. That these deposits constitute evidence of intense tropical cyclone strikes is further demonstrated by the distribution of recent coarse deposits within Muyil (Figure S11). A multi-decadal (20-year) count of coarse beds present in the well preserved C1 core-top positively correlates with an identical count of historically observed major hurricanes passing within 100 km of the cenote (r = 0.58, *p* < 0.05). The correlation increases to r = 0.70 (*p* < 0.05) if we only consider the more recent 60 years of the observation record when airplane reconnaissance and satellite monitoring improved observational accuracy^36,37^. If coarse deposition occurred independently from tropical cyclone activity, we would not expect to see a correlation of any sort between modern deposition and historically observed storms. The fact that a correlation exists at all introduces confidence that coarse deposits reflect near-to-direct strikes from major hurricane events.

## Discussion

### Hurricanes and droughts

**Figure 4:**
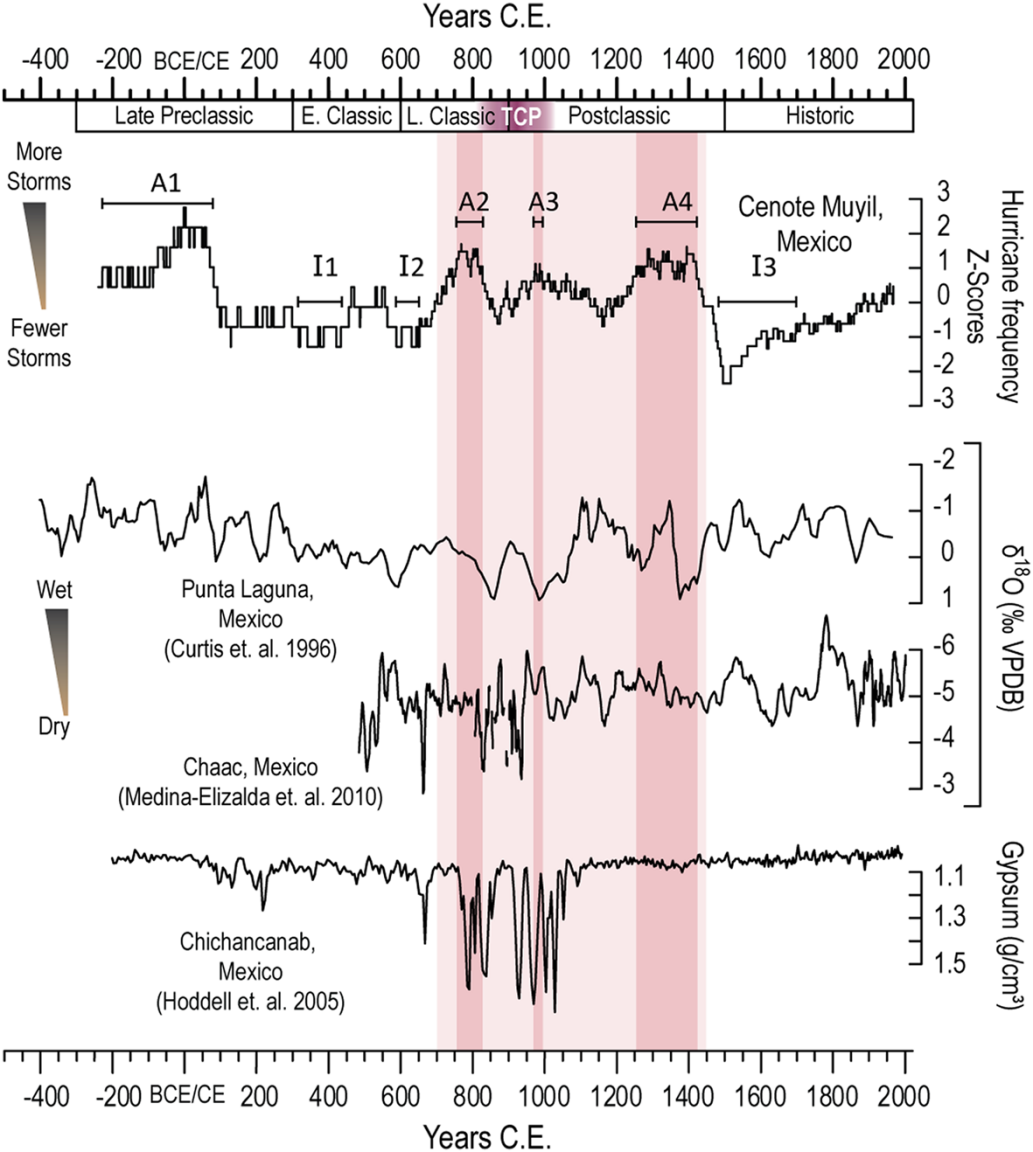
Top: Centennial-scale hurricane frequency (shown as Z-Scores depicting activity above or below the mean) from combined sediment record from Cenote Muyil, along with statistically significant intervals of active (A1 to A4) and inactive (I1 to I3) storm occurrence. Pink shading highlights persistent period of above average hurricane activity from 700 to 1450 CE. Dark pink bands depict the anomalously active periods falling within that window. Middle: δ^18^O measurements on ostracod valves from Punta Laguna ^11^ and the Chaac speleothem ^38^. More depleted values are nominally indicative of increased rainfall. Bottom: Gypsum concentrations from Laguna Chichancanab ^16^, whereby high gypsum concentrations are thought to indicate intervals of increased aridity.

Unsurprisingly, the centennial variations in coarse deposition inconsistently correlate with geographically proximal records of regional water balance over the Common Era. Active intervals A1 and A2 are concurrent with depleted δ^18^O values in lacustrine ostracod valves recovered from Punta Laguna ^11^ (Fig. 4), nominally indicative of wetter conditions. A3 (975 to 985 CE) is contemporaneous with more enriched δ^18^O values from Punta Laguna, which would suggest increased local evaporative forcing and aridity^11^, however the δ^18^O value for calcite from the Chaac speleothem in the northern Yucatan^38^ suggests wetter conditions at this time. This disagreement between the Punta Laguna ostracods and Chaac speleothem δ^18^O records may result from dating uncertainties, proxy sensitivity, seasonality of proxy generation, and time-averaging. Muyil interval A4, from 1285 to 1420 CE, occurs during periods of both δ^18^O enrichment and depletion in Punta Laguna. The anomalously quiescent interval I3 (~ 1500 to 1750 CE) coincides with evidence for both wetter and drier conditions from Punta Laguna and the Chaac speleothem. Apparent contradictions between the hurricane and precipitation records may (1) reflect sensitivity in the isotopic record to synoptic changes in atmospheric circulation, moisture source, or precipitation type rather than direct rainfall amount^21,39–41^, or (2) reflect the modern lack of positive correlation between intense hurricane frequency and precipitation on multidecadal timescales (Figure S1). This lull in hurricane activity during Muyil interval I3 however, does coincide with a series of historically observed droughts initiating in the sixteenth century ^42^ suggesting that both regional precipitation and hurricane activity were suppressed during portions of the Little Ice Age from ~ 1400 to 1800 CE ^38^.

In addition to these δ^18^O records, variable gypsum deposition within Laguna Chichancanab in the central Yucatan has been used to identify local hydroclimate shifts between ~ 650 and ~ 1100 CE^16^. Gypsum forms as a precipitate within sulphate-enriched groundwater when periods of decreased rainfall and increased evaporation favor the chemical kinetics of sulphatic-mineral precipitation. The Chichancanab record indicates multi-decadal droughts disrupted by brief pluvial periods during the TCP. Intervals A2 and A3 are both contemporaneous with these highly variable gypsum transitions, though the temporal resolutions of the individual records prevent precise correlation.

The paleo-hurricane records most proximal to Muyil are the multiple reconstructions from Great Blue Hole on Lighthouse Reef, Belize^43–45^ (Figure S12). On centennial timescales, the new reconstruction from Cenote Muyil broadly corresponds to the patterns of storm activity identified in Gischler et al.^44^ and Denommee et al.^43^, which all show increased activity during the Late and Postclassic Periods and decreased activity during the Little Ice Age. Furthermore, the recent work of Schmitt et al.^45^ does acknowledge evidence of tropical cyclone activity coincident with arid intervals in the ninth and eleventh centuries. It remains a problem, however, that the reconstruction of Schmitt et al.^45^ substantially diverges from the earlier records from Great Blue Hole (see Supplementary Figure S12). Confoundingly, the earlier and more recent reconstructions depict very different storm frequencies over the past millennium. While chronostratigraphic uncertainties in the reconstruction presented by Denommee et al.^43,45^, may have contributed to these disagreements, the differences in these reconstructions have not yet been resolved and render comparisons between the records from Muyil and Great Blue Hole uncertain. Independent of this uncertainty, reconstructions from Belize may not accurately characterize the storm climate experienced by the Classic Maya in the north considering that the northern Yucatan Peninsula experiences a much a higher proportion of hurricane strikes (72%) relative to the south. Hurricane reconstructions from northern sites like Cenote Muyil offer better insight into the conditions confronting the Northern Lowland Maya during key cultural intervals.

### Maya cultural implications

The most striking feature from this record is that centennial storm frequency was above the mean for 610 of the 750 years (81%) between 700 and 1450 CE, an interval coincident with multiple and significant realignments of the Classic and Postclassic Lowland Maya. While the lower temporal resolution of the sedimentological record may preclude correlations between individual storm occurrences and specific historical events, the multidecadal frequency analysis does characterize the background hurricane conditions that would have been experienced by regional populations. The increase in storm activity ~ 700 CE coincides with shifting demographics as southern populations migrated northward^5^ perhaps motivated by local water scarcity. The effects of drought would have been felt most acutely in the southern and central lowlands^17,46^ where the more elevated terrain would have limited access to potable groundwater. In the low-lying northern Yucatan, groundwater would have remained accessible during intervals of diminished rainfall. This may have helped buttress northern polities during the initial stages of aridity and aided the continued growth of cultural centers such as Chichén Itzá and Cobá^1^. However, the combination of persistent drought and increased storminess in the north may have contributed additional stress among local populations^47^ since drought conditions can amplify storm damage and further exacerbate agricultural loses^48,49^. This relationship was directly observed when crop losses driven by a Yucatan landfalling hurricane in 1772 CE were prolonged by the onset of drought conditions and the arrival of locusts the following year^50,51^. Furthermore, storm-related agricultural losses and subsequent food supply destabilization may persist for several years following a hurricane event^52–54^ such that even minor increases in local storm activity may lead to compounding losses over time.

While the northern Postclassic Maya experienced degrees of economic development and prosperity not observed in the central lowlands^18,19,30^ this period also contained instances of political collapse, demographic reconfiguration, and violence^31,55–57^. This work neither suggests that any single storm may have been a direct causal factor for concurrent cultural events, nor presents hurricanes as a cause-and-effect linkage between contemporaneous societal challenges. However, the new hurricane reconstruction provided here demonstrates that widespread societal disruptions, realignments, and economic growth occurred synchronously with evidence for increased northern Yucatan hurricane activity. The Postclassic expansion of maritime trade may speak to the greater resource availability offered by seaborne commerce as well as the resiliency and economic value of coastal centers (such as Muyil)^18^. The onset of anomalously high storm frequency ~ 800 CE is contemporaneous with a significant decrease in the number of dated stone inscriptions found throughout Maya territory, as well as subsequent abandonment of the Maya Long-Count calendar^11,58^. The decline of Chichén Itzá in the eleventh century^1^ occurs within this extended period of above average storminess, but does not clearly align with any of the anomalously active intervals. However, the decline of Cobá in the thirteenth century^59^ is coincident with active interval A4 (1285 CE to 1420 CE) (Fig. 3) which may indicate a cultural response to increased ecological strain. While the emergence of coastal cities (e.g., Tulum) and the rise of Mayapán as a regional power center demonstrate a Postclassic Maya resurgence^18^, these advances do not negate the persistence of social and environmental stresses^6,31,55,57^. Both Tulum and Mayapán were walled cities, denoting an increased focus on militarism and urban defense^55,56^. Active interval A2 (755 CE to 825 CE) is concurrent with an increase in inter-polity warfare as derived from dated references to war-related activities^6^ (Fig. 3). Evidence of conflict at Mayapán, including mass graves^55,56^, coincides with active interval A4. This is consistent with surviving records noting that convergent hazards, including drought, famine and hurricanes, impacted Mayapán throughout the fourteenth and fifteenth centuries^50,54^. Natural disasters may lead to violent civil conflict^60^. The combination of recurrent drought and elevated hurricane frequency could have intensified issues of security and encouraged hostility as well as adaptive strategies.

Extant Maya accounts, such as those recorded in the Book of Chilam Balam ^61^, describe the impact major hurricanes had on the region. The “hurricane of the four winds” stuck the Yucatan in 1464 CE^54^, was subsequently documented by the Spanish Bishop Diego de Landa in the early 1500’s, and reportedly destroyed structures, injured locals, and denuded the landscape of vegetation and game^62^. Though the landfalling location of this storm is not recorded, this event is concomitant with the anomalous Sl-6 slump feature and associated hiatus, which is suggestive of a naturally occurring extreme event *(Supplemental Text S2)*. While the extent that heightened hurricane frequency may have contributed to societal disruption or adaptation is unknown and requires further academic inquiry beyond the scope of this work, it is clear from the surviving Maya records that storms occupied an important position on the Maya landscape. The culture was acutely aware of the potential devastation wrought by these events and as such, regional hurricane variability should not be ignored when exploring the changing demography of the Classic and Postclassic Maya.

## Conclusion

The sedimentological archive from Cenote Muyil shows that the Northern Maya lowlands were subjected to above average hurricane activity for much of the Late Classic and Postclassic Maya Periods, including the Terminal Classic Phase. Given the differential meridional susceptibility of the Yucatan Peninsula to storm strikes, this work provides regional specific insight into the potentially devastating conditions experienced by the northern Maya communities during this important historic interval. The Postclassic period was a time of cultural reconfiguration in the northern Yucatan owing to depopulation of the southern and central lowlands, an increase in maritime trade, the collapse of previous power centers and the emergence of new ones. Increased hurricane frequency in the north could have posed an additional environmental variable that should be considered when discussing the Maya political, economic, and architectural transformations at the time. It is all but certain that no single factor bears absolute culpability for the centuries long realignment of the Classic Maya. The cause of their decline was likely multivariate involving both external and internal pressures. Drought, political divisions, and shifting demographics may have all stressed the northern Maya as they weathered these Terminal Classic tempests.

## Materials and methods

Two continuous 7.6 cm diameter sediment cores were collected from Cenote Muyil using a Rossfelder P-3 submersible vibracore system deployed from a portable coring platform suitable for shallow environments. Cores were sectioned into 150 cm lengths in the field prior to transportation to Texas A&M Galveston for analysis. Downcore textural variability was quantified using a modified Sieve-First Loss-On-Ignition procedure in which contiguous 2.5 cm^3^ sediment samples were wet sieved through a 63 μm mesh to remove fine-grain (< 63 μm) particles prior to combustion. Sediment deposits were categorized as tempestites if their coarse fraction value surpassed the 95th percentile along an 11-point moving mean once the background sediment signal had been removed. For a more complete discussion of statistical and laboratory analyses see *Methods* in Supplemental text S1.

## Supplementary Information

Supplementary Information.

## Data Availability

Datasets generated this research (grain size, chronology) are available from the National Oceanic and Atmospheric Administration’s National Centers for Environmental Information (NCEI) paleoclimatology repository (https://www.ncei.noaa.gov/access/paleo-search/study/36998).
