# Author Correction: Northeast Yucatan hurricane activity during the Maya Classic and Postclassic periods

**DOI:** 10.1038/s41598-023-28718-6

**Published:** 2023-01-30

**Authors:** Richard M. Sullivan, Peter J. van Hengstum, Jeffrey P. Donnelly, Anne E. Tamalavage, Tyler S. Winkler, Shawna N. Little, Luis Mejia-Ortiz, Eduard G. Reinhardt, Sam Meacham, Courtney Schumacher, Robert Korty

**Affiliations:** 1 Department of Oceanography, Texas A&M University, College Station, TX 77843 US; 2 Department of Marine and Coastal Environmental Science, Texas A&M University at Galveston, 1001 Texas Clipper Road, Galveston, TX 77553 US; 3 Geology & Geophysics, Woods Hole Oceanographic Institution, 266 Woods Hole Rd, Woods Hole, MA 02543 US; 4 Département de Sciences Biologiques, Université de Montréal, 1375 Avenue Thérèse-Lavoie-Roux, Montréal, QC H2V 0B3 Canada; 5 Department of Marine Biology, Texas A&M University at Galveston, 1001 Texas Clipper Road, Galveston, TX 77553 US; 6 División de Desarrollo Sustentable, Universidad Autónoma del Estado de Quintana Roo, Av. Andrés Quintana Roo s/n, 77600 Cozumel, Quintana Roo Mexico; 7 School of Geography and Earth Sciences, McMaster University, Hamilton, ON L8S 4K1 Canada; 8 CINDAQ - El Centro Investigador del Sistema Acuífero de Quintana Roo, A.C., Puerto Aventuras, Quintana Roo Mexico; 9 Department of Atmospheric Sciences, Texas A&M University, College Station, TX 77843 US

Correction to: *Scientific Reports* 10.1038/s41598-022-22756-2, published online 22 November 2022

The Supplementary Information file published with this Article contained errors.

The spelling of the author Luis Mejia-Ortiz was incorrectly given as Luis Mejita-Ortiz.

Additionally, Affiliations 1, 3, 6 and 9 were incorrect.

Affiliation 1 was incorrectly given as ‘Department of Oceanography, Texas A&M University, Bizzell St, College Station, TX 77840’. The correct affiliation is listed below.

Department of Oceanography, Texas A&M University, College Station, TX 77843

Affiliation 3 was incorrectly given as ‘Geology & Geophysics, Woods Hole Oceanographic Institution, MS #22, 266 Woods Hole Rd, Woods Hole, MA 02543’. The correct affiliation is listed below.

Geology & Geophysics, Woods Hole Oceanographic Institution, 266 Woods Hole Rd, Woods Hole, MA 02543

Affiliation 6 was incorrectly given as ‘División de Desarrollo Sustentable, Universidad de Quintana Roo, Blvd. Bahía s/n, Del Bosque, 77019 Chetumal, Q.R., Mexico’. The correct affiliation is listed below.

División de Desarrollo Sustentable, Universidad Autónoma del Estado de Quintana Roo, Av. Andrés Quintana Roo s/n, 77600, Cozumel, Quintana Roo, Mexico

Affiliation 9 was incorrectly given as ‘Department of Atmospheric Sciences, Texas A&M University, Bizzell St, College Station, TX 77840’. The correct affiliation is listed below.

Department of Atmospheric Sciences, Texas A&M University, College Station, TX 77843

These errors have now been corrected in the Supplementary Information file that accompanies the original Article.

