# Supplementary material for: Northeast Yucatan hurricane activity during the Maya Classic and Postclassic periods
Source: Sci Rep. 2022 Nov 22;12:20107. doi: 10.1038/s41598-022-22756-2 (PMC9684114; doi:10.1038/s41598-022-22756-2)
Supplement: Supplementary file 1 — Supplementary Information. [file 41598_2022_22756_MOESM1_ESM.pdf]

Supplementary Information for
Northeast Yucatan Hurricane Activity During the Maya Classic and Postclassic Periods

Richard M. Sullivan[1], Peter J. van Hengstum[1,2], Jeffrey P. Donnelly[3], Anne E. Tamalavage[1,4], Tyler S. Winkler[1,3], Shawna N. Little[5], Luis Mejia-Ortiz[6], Eduard G. Reinhardt[7], Sam Meacham[8], Courtney Schumacher[9], Robert Korty[9]

1. *Department of Oceanography, Texas A&M University, College Station, TX 77843; *richardmsullivan@tamu.edu*
2. *Department of Marine and Coastal Environmental Science, Texas A&M University at Galveston, 1001 Texas Clipper Road, Galveston, TX 77553*
3. *Geology & Geophysics, Woods Hole Oceanographic Institution, 266 Woods Hole Rd, Woods Hole, MA 02543*
4. *Département de Sciences Biologiques, Université de Montréal, 1375 Avenue Thérèse-Lavoie-Roux, Montréal QC H2V 0B3*
5. *Department of Marine Biology, Texas A&M University at Galveston, 1001 Texas Clipper Road, Galveston, TX 77553*
6. *División de Desarrollo Sustentable, Universidad Autónoma del Estado de Quintana Roo, Av. Andrés Quintana Roo s/n, 77600, Cozumel, Quintana Roo, Mexico*
7. *School of Geography and Earth Sciences, McMaster University, Hamilton, ON, L8S 4K1, Canada*
8. *CINDAQ - El Centro Investigador del Sistema Acuífero de Quintana Roo, A.C., Puerto Aventuras, Mexico*
9. *Department of Atmospheric Sciences, Texas A&M University, College Station, TX 77843*

Corresponding author: Richard M. Sullivan
Email: richardmsullivan@tamu.edu

This PDF file includes:

Supplementary text (S1 to S4)
Figures S1 to S14
Tables S1 to S3
SI References

Supplementary Information Text

This supporting information provides additional details about methods, analysis, and discussion of related sedimentology relevant to this work. Additional figures and tables are included that may assist the reader in interpreting this analysis.

S.1. Methods

S1.1. Sediment Core Collection

Previous work has documented that sedimentary processes in individual terrestrial sinkholes are not always archived uniformly due to basin specific geometries. This necessitates a multi-core approach to fully understand sedimentary dynamics in these unique places [1-3]. In 2017 two continuous sediment cores (7.62 cm diameter) were collected from 17 m depths (**Figure S2**) using a Rossfelder P3 submersible vibracore system from a portable coring platform suitable for working in shallow environments typically inaccessible by deeper draft vessels. Core 1 captured 12.1 meters while faithfully preserving the sediment water interface. Core 2 recovered 12.5 meters, though the sediment water interface was not as well preserved as in Core 1. Core recovery was limited by the length of available core barrels since the bottoms of each core showed no indication of contact with a hard/impenetrable substrate. Given these observations, it is unlikely that the entire Holocene sequence was recovered, and additional sediment potentially remains to be sampled from Cenote Muyil. Cores were sectioned into 150 cm lengths in the field for transport to Texas A&M Galveston where they were subsequently split, photographed, and X-radiographed.

S1.2. Textural Analysis

Downcore textural variability was quantified using a modified Sieve-First Loss-On-Ignition procedure [4, 5], which is well suited for evaluating changes in coarse particle deposition in lake-like settings on karst landscapes (e.g., sinkholes, blue holes). 2.5 cm^3 subsamples were contiguously extracted at 1 cm intervals from all core sections. After sampling, sub-samples were wet-sieved through a 63 μm mesh to remove fine-grain (<63 μm) particles while concentrating the coarser fraction. Samples were dried over-night at 80º

C, weighed, and then combusted for 4.5 hours at 500º C to remove any remaining organic matter. Post-ignition masses are used to quantify downcore changes in coarse grain particle deposition, and are expressed in mass per unit of volume ($D_{>63}$ μm mg/cm^3). Ratios of total organic carbon to total nitrogen (C:N) were measured on downcore bulk sediment samples to characterize any past changes in organic matter source [6]. Sample spacing was approximately 30 to 60 cm, however sediment was sampled at higher spatial resolutions around coarse deposits or sedimentary features. For further discussion see *Supplemental Text S4*.

S1.3. Radiocarbon Dating and Age Model Development

Age-depth models were developed from plant fragments (e.g., seeds and leaves, *n = 13*) that were radiocarbon dated at the National Ocean Sciences Accelerator Mass Spectrometry (NOSAMS) facility at the Woods Hole Oceanographic Institution. Terrestrial plant macrofossils provide the best age estimate for a given stratigraphic interval since plants from terrestrial environments incorporate radiocarbon exclusively from the atmospheric carbon pool. Conventional radiocarbon results arising from plant macrofossils were calibrated into calendar years using IntCal20 [7]. Downcore age models for each core were developed using a Bayesian statistical approach within the Bacon v2.5 software package operated in the R computing environment v4.0.3 [8]. Twenty-four radiocarbon dates were initially extracted from the two cores, however 11 were excluded from the age model since they did not depict a coherent age-depth relationship consistent with their stratigraphic order.

All radiocarbon measurements on organic matter fragments provided calibrated age results that are less than ~2200 years old, suggesting the recovered successions accumulated during the Common Era (CE). However, while 24 radiocarbon dates were extracted from the

two cores (15 from C1, and 9 from C2; **Figure 2**), only 13 followed a stratigraphically consistent chronology. The remaining 11 were all older than could be reasonably expected for the depth from which they were recovered. Anomalously old ages can sometimes be attributed to reworked older material (e.g., the 760 ± 15 BCE date from 1050 cm in C2), or material that is impacted by hardwater effects. While care was taken to ensure that only terrestrial plant remains were selected for AMS dating, not all the material extracted for this purpose could be positively identified as derived from the terrestrial surface (e.g., seeds, leaves). As such, it is possible that unknown plant fragments extracted from the upper part of the cores came from submerged aquatic vegetation and were impacted by a hardwater effect. This is supported by the more enriched $\delta^{13}C_{org}$ values of radiocarbon results near the core-tops **(Figure S13)**, which may be more indicative of an aquatic carbon source [9]. Six of the upper inconsistent dates fall between 100 ± 30 CE and 600 ± 40 BCE, which makes them ~2000 years older than the estimated ages for their depths based on the position of younger age results.

It is important to note that ten of the 11 inconsistent dates were extracted from within Phase 1 of the two cores (seven from C1, and three from C2). While these dates may have been older reworked material, it also possible that these upper dates were impacted by hard-water effects from in situ produced organic material. The low C:N values present in Phase 1 (*See Supplemental Text S4*) may reflect an increase in primary productivity [6, 10] that contaminated organic material submitted for AMS dating. The reduction in storm activity during the Little Ice Age likely minimized the transport of terrestrial organic matter into the cenote meaning that most organic material present within the core between ~1450 to 1800 CE had an aquatic provenance. Both ^{210}Pb and ^{137}Cs were similarly unable to provide

confident chronographic horizons within Phase 1, perhaps due to poor adherence of the radioisotopes to the carbonate sedimentary particles. However, AMS dates from Phases 2 and 3 are considerably more chronologically consistent.

To ensure chronological parity of the records, stratigraphic tie-points were selected by tracing clear sedimentary contacts between cores 1 and 2 which were assumed to be contemporaneous. Two tie-points were identified within C1 and C2 bracketing a prominent sedimentary feature clearly discernable in each core (**Figure 2**). This feature presents as a slump in C1 (identified as Sl-6) and a corresponding hiatus in C2 (**Figure 2, S6**). The uppermost tie point (H1; **Figure 2**) was extracted from a coarse bed directly above the slump in C1 (304 cm depth in core). The mean Bacon derived age of 1568 CE was applied to the corresponding contact immediately above the hiatus in C2 (263 cm depth). The second tie point, H2, was a coarse bed directly at the base of the slump in C1 (425 cm). The mean Bacon derived age of 1444 CE was applied to a coarse contact at the base of the hiatus in C2 (271 cm). A third tie point, H3, was identified at a depth of 941 cm in C1 and 713 cm in C2. This feature was a narrow coarse deposit situated between multiple successive slump features that were each traceable between the two cores. A radiocarbon date extracted from this coarse bed in C2 yielded the uncalibrated AMS date of 750 ± 15 CE. The age was applied to the corresponding coarse contact in each core. The tie points bracketing the upper slump/hiatus feature ensured that each age model coherently depicted the timing of this feature. The lower tie point (H3) minimized older sediment emplacement age disagreement between the cores possibly arising from the variable width of the older slump features. Omitting the tie points produced an asynchronous chronology between C1 and C2 for features that are stratigraphically synchronous.

S1.4. Event-bed Identification

Given the lack of classic riverine inputs to the study site, abrupt increases in coarse grained deposition are most likely related to abrupt re-suspension and initial settling-out of coarse sedimentary particles following an increase in wind and wave energy from an intense storm event (e.g., Brown, Reinhardt [2]), such as a hurricane or winter storm. Sediment samples from the shallow fringing margin of the lagoon were predominately sand sized (> 63μm diameter) particles indicating a likely sediment source during storm passage. Hurricane force winds traveling from the northeast along the lagoon's long axis would generate significant internal waves. Coarse sediment entrained by these waves would be deposited within the cenote. Observed coarse beds are not shell beds (either ostracodes, gastropods, or bivalves), which precludes an invertebrate-based ecologic explanation. In fact, very little shell material was recovered at all in the stratigraphy. To interpret these event-driven coarse beds they must be considered separately from the background autogenic carbonate-driven sedimentation. Identifying the likely background sedimentary signal required first collapsing all contiguous anomalous deposits of assumed instantaneous deposition greater than 5 cm in thickness (**Figure 2, S6, S7**) to single 1 cm thick beds. Next, a five-point running mean was used as a first pass filter to identify and remove obvious peaks from coarse fraction data. Peaks were omitted from the record if they exceeded the mean value of that five-point running average. Resulting gaps were then linearly interpolated before the record was again smoothed using an 11-point moving mean to identify the background sediment signal and minimize decadal-scale variability in autogenic carbonate

deposition. This background signal was subtracted from the raw grain size data to generate the coarse-anomaly record.

While the most significant coarse deposits likely resulted from anomalously high depositional energies, it is unlikely that all coarse beds exceeding background sedimentation represent clear evidence of near-to-direct striking storm events. Minor variations in coarse particle deposition may result from internally driven basin processes (i.e. biogenous sediment such as calcite tubules that may develop around aquatic vegetation). To isolate coarse deposits indicative of near-to-direct striking storm events we first calculated the standard deviations of the coarse-only signal using an 11-point moving window. Deposits were deemed significant event beds if their coarse fraction value surpassed the 95th percentile for that interval and if that value exceeded the mean coarse fraction of the two adjacent intervals by 50%. This second step was required to avoid overcounting in intervals with highly variable background sedimentation that could not be completely accounted for (such as through large scale sedimentary shifts). The record was then manually adjusted in accordance with the following criteria. If two or three successive 1 cm intervals were categorized as significant, the depth of the maximum value within that couplet or triplet was assigned to the event and the other interval(s) were grouped with that event. For continuous coarse deposits exceeding 3 cm in width, peaks separated by at least 2 cm of decreasing values were assumed to be independent events. This method of distinguishing event beds differs from those used in other coastal karst environments [11, 12]. The long-term decrease in mean event bed density within Cenote Muyil meant that those methods were inappropriate as they assume a degree of consistency among all event beds within a sediment archive.

Event bed count were summed over a 100-year moving window to quantify centennial-scale event distribution. The separate frequency counts were each scaled to their standard z-score which reports how many standard deviations each observation is from the mean. Finally, the separate standardized records were combined into a single composite record for the cenote. Individual coarse beds may not be recorded in both cores due to issues of internal basin geometry and/or the energy and direction of the depositional event. Deposits present in one core but not the other may indicate under-sampling within an individual core or over-sensitivity (and thus over-sampling) within the other. To resolve this, the standardized records for the overlapping portions of the two cores were averaged into a single record showing standard deviations around the mean return interval.

S.2. Slump-like Deposits

Of the 15 (11) slumps present in C1 (C2), nine (five) display clear fining upward sequences. These thicker deposits may have been produced by slow moving tropical storms driving continuous mobilization of the grains fringing the edge of the cenote. However, gravity driven sediment flow resulting from slope failure on the sides of the hole cannot be ruled out. Kenter [13] found that slopes dominated by fine-grained carbonate sediments have angles of repose rarely surpassing 15°. The northwest slope of Cenote Muyil is currently ~10° **(Figure S2)** and may not require an external mechanism to initiate a slope failure. These sides of the hole may have undergone periodic collapse if the slope angle approached the limit of internal stability. Slump Sl-6 in C1 and the corresponding hiatus in C2 are the features one may expect if gravity driven sediment flow is the mechanism. While slope failures may be induced by tectonic instability, impacts of seismic activity on the northern

Yucatan Peninsula are not well documented. Considering that the three most recent slump deposits occurred in the last 200 years and that there are no earthquakes mentioned in any historical records, it is unlikely that these features were seismically produced. However, minor tremors (3 on the Richter scale) were felt in Quintana Roo and reported by local news outlets following a 7.7 magnitude earthquake that occurred off the west coast of Jamaica in 2017. Minor seismic events could potentially pulse ground water systems and disturb cave or cenote sediments, though no published accounts from the Yucatan exist at this time to confirm this.

The remaining slump features (six within each C1 and C2) display mixed or coarsening upwards sequences. Such sequences may represent **i)** consecutive storms leaving overwash deposits in rapid succession absent a return to background sedimentation, **ii)** increased depositional energy from storm backflow following storm passage [14, 15], or **iii)** a prolonged storm-driven wave system such as a seiche. The larger coarse horizons at the base of several of the slump deposits (C1: Sl-9, Sl-10, Sl-11, Sl-15; C2: Sl-10, Sl-11) may indicate an initial passing storm followed by prolonged basin-wide sloshing and sediment resuspension and transport [16].

Seiches are oscillatory waves that may form within semi-enclosed basins [17, 18]. A persistent standing wave oriented along the transverse axis of the lagoon could potentially generate the sustained energy needed to produce these thicker deposits. Seiches can result from earthquakes or tangential wind-stress and may mobilize or resuspend bottom sediments for deposition in the cenote. Additionally, lake seiches may be produced by hurricane driven winds [19, 20], though the development of the seiche may depend on the orientation of the passing storm and not all storms may generate seiches within the lagoon. First order wave

height calculations [21] show that in a fetch-limited basin such as Chunyaxché (7 km along the long axis) significant wave heights may exceed 3.6 m during wind conditions comparable to a Category 3 hurricane strike (sustained winds > 96 kt). Wave heights may reach 6.0 m when exposed to sustained Category 5 wind speeds such as those generated by Hurricane Gilbert in 1988.

The disparity in slump thickness and shape between the two cores may be the result of core location within the hole and the fining of the deposits across the basin. C1 is closer to the center of the cenote and is proximal to the gentler slope (~6°) of the southeastern sides (**Figure S2)** and C2 is closer to the steeper northwest side of the hole (~10°). Thicker deposits in C1 may be indicative of sediment transported from the east or south of the hole that fine toward the northwest. The possible density driven flow that produced Sl-6 may have originated on the steeper northwest slope closer to C2. Slope failure on the steeper side of the hole may have been more likely to scour the C2 location and transport the material towards C1.

S.3. Sedimentary Phases

The mechanisms behind the distinct changes in mean coarse bed density occurring within the three sedimentary phases are unknown. However, they may be related to baseline changes in depositional energy (i.e., stronger storms) or changes to sediment supply within the lagoon system. While we cannot discount changes to past storm intensity, we may consider that changing environmental conditions over the centuries could have altered the adjacent mangrove wetland surrounding Laguna Chunyaxché or promoted an increase in fine grain marl sedimentation directly through internal basin process. Considering the oligotrophic and phosphorus-limited nature of the ground water within the Sian Ka’an

reserve [22], it is unlikely that the wetland underwent periods of continued expansion, but persistent aridity during the Early, Classic, and Postclassic periods may have encouraged growth of the more drought tolerant fringing mangrove species *C. erectus* [23] which favors the supratidal zone. A return to generally wetter conditions in the late Postclassic [24] would have encouraged the growth and dominance of *R. mangle,* which is common in the wetland today. Continued dominance of subtidal mangrove species would have increased sediment retention along the lagoon edge, inhibit the re-entrainment of settled particles, and limited coarse particle mobilization [23, 25-28]. Additionally, mangroves have a baffling effect on local flow which may inhibit the transport of coarser grains during periods of increased hydrodynamic activity. Mangrove driven siltation was also identified as the probable cause for the narrowing and infilling of lagoonal waterways at the coastal Mayan site of Vista Alegre in the northeast Yucatan [29, 30].

Reductions in precipitation would not have driven decreases in lake levels through evaporative processes since surface water elevation is mediated by local sea level [31, 32] and the lagoon is replenished through the subsurface aquifer. Regional sea levels have been relatively stable during the Common Era and exhibited only gradual rise [33], though even modest increases in water level may alter the sensitivity of a basin and promote local changes in sedimentation. Increased mixing of thermally distinct groundwater masses (perhaps driven by minimal sea level change) may have resulted in CO_2 degassing of the alkaline and calcium rich waters. This process would have continually encouraged fine-grain lacustrine marl precipitation within the basin. If this process was driven by gradual sea-level rise, then we would expect a continuous progression of finer background sediment over the Common Era such as that observed in the cores. It is likely that a combination of these

factors (increased mixing of water masses, changes to shoreline vegetation, and increased sediment baffling) dampened coarse grain transport over time, with the most pronounced change occurring ~1450 CE.

Further evidence for increased lagoon siltation was observed in the archaeological record [34]. The Mayan sacbes (causeways) were likely used by the occupants of Muyil to facilitate cargo transport between canoes and local storage facilities. However, the sacbe network currently terminates 140 m landward of the modern Laguna Muyil shore. While the timing of the original network construction is uncertain [34] sections of the sacbes closest to the modern lagoon edge were most likely constructed in the Late Postclassic period as extensions to the older causeways. This suggests that the shoreline expanded into the lagoon during the Mayan occupation and may have required periodic extension of the causeways to maintain access.

S.4. Carbon / Nitrogen

Ratios of Total Organic Carbon (TOC) to Total Nitrogen (TN) reflect a terrestrial or aquatic source of sedimentary organic matter. C:N values <10 are indicative of aquatic organic material (i.e. freshwater algae) and values >20 reflect a terrestrial provenance. Intermediate values suggest mixed input [6]. Organic Carbon (OC) values were obtained by first digesting bulk sediment with 10% HCl for 24 hours. Treated samples were rinsed with deionized water, then dried and pulverized. Samples for Total Nitrogen (TN) were untreated and just dried and homogenized. OC and TN (recorded as percent mass) were analyzed using ~3 mg of dried samples on a Costech Elemental Analyzer. Acetanilide was used as a standard reference material following the National Institute of Standards and Technology

guidelines for calibrating marine sediments. C:N was calculated from the acidified carbon and nitrogen fractions.

Core 1 and Core 2 each exhibit changes in the ratio of atomic carbon to nitrogen over time that are broadly consistent with the three distinct sedimentary phases **(Figure S14)**. Measurements taken from C2-Phase 3 ($n = 6$) have a peak C:N value of 13.35 and a mean of 12.30 **(Table S2)**. C2-Phase 2 ($n = 13$) decreases to a mean of 10.82. C2-Phase 2 has a maxima of 12.75 and a minimum value of 8.71 that both occur in the upper 60 cm of that phase at (~1330 and 1380 CE respectively). C2-Phase 1 ($n = 7$) can be further subdivided based on the C:N results. The older intervals of C2-Phase 1 ($n = 4$) have a mean C:N value of 9.49, and a mean of 11.53 during the 20th century ($n = 3$). We see a similar trend of gradual decrease over time in C1 despite the C:N values being consistently higher than in C2. Phase 2 in C1 ($n = 20$) has a mean of 12.95. The maximum value of 15.35 occurs at 730 CE and the minimum value of 10.383 at 646 CE. However, the next lowest value of 10.45 occurs in 1394 CE, which is temporally proximal to the minima in C2-Phase 2. C1-Phase 1 ($n = 21$) is more varied than C2, though this may be due in part to the larger sample number. These results indicate that organic material in the lagoon sediments was primarily aquatic in origin with limited terrestrial input.

Supplementary Figures

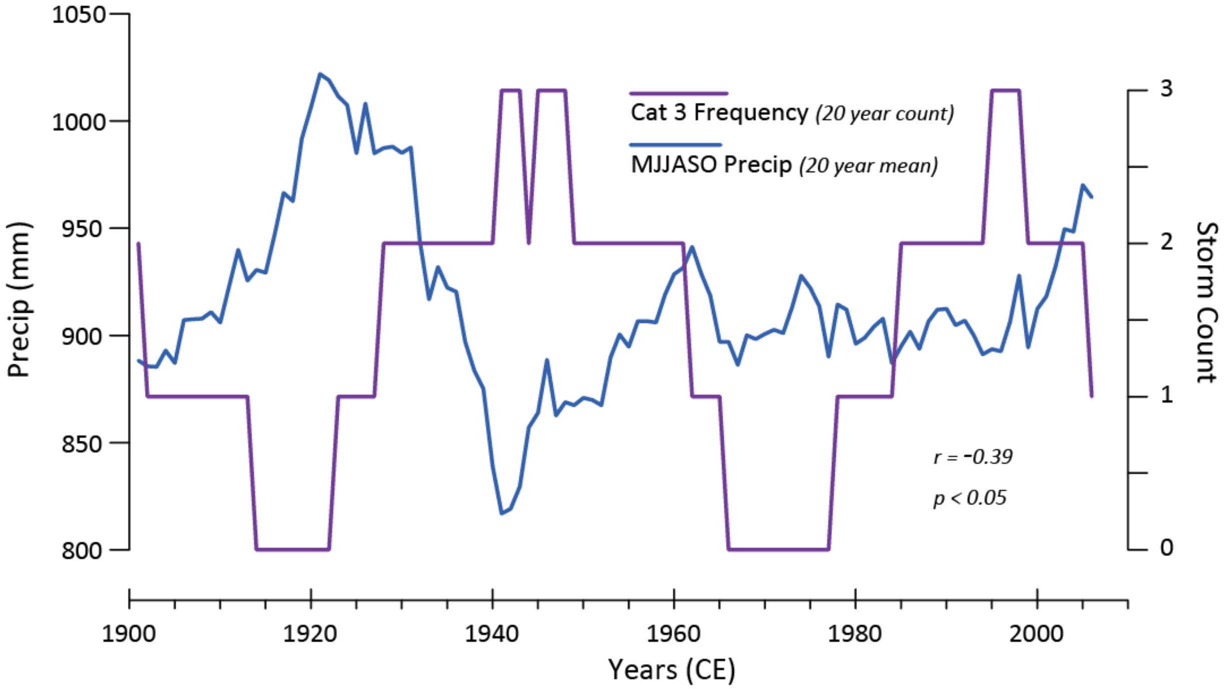

Figure S1. Comparison of modern rainfall totals and major hurricane passage within 100 km of Cenote Muyil. Multi-decadal (20-year) relationship between wet season rainfall (blue) and hurricane frequency (fuchsia) shows a low correlation. This indicates that time-averaged records of rainfall are insufficient proxies for tropical cyclone frequency. Monthly total rainfall data was extracted from 11 0.5° x 0.5° Global Precipitation Climatology Centre v2018 [35] grid cells that fell within 100 km of Cenote Muyil. Grid cells were only included if at least 50% of the land covered by the cell fell within the 100 km radius. Storm counts came from NOAA's best track historical database [36].

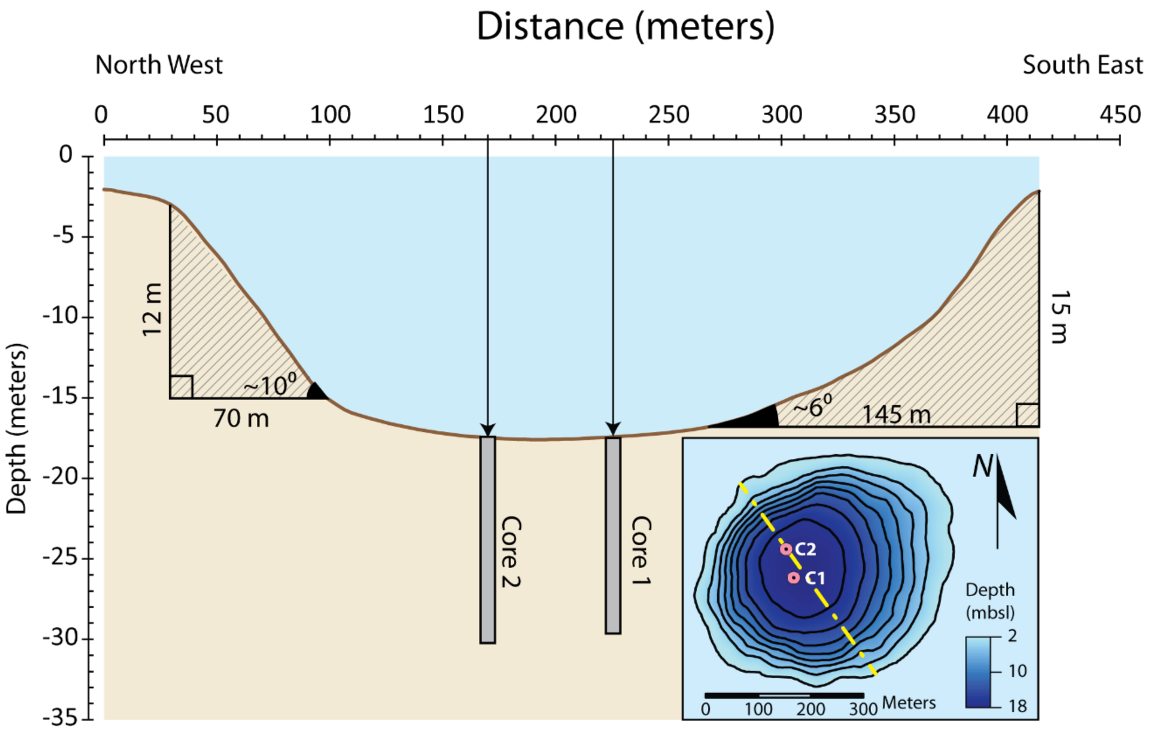

Figure S2. Cross section of Cenote Muyil depicting core locations and mean slope angles of the northwest and southeast slopes. Note that the northwest slope adjacent to C2 has a higher angle of repose (~10°) than the slope nearest C1. The inset shows the bathymetric model with core locations. Yellow line indicates cross section shown. Bathymetric surveying was conducted using a Hydrolite-DFX dual frequency (200/30 kHz) single beam echosounder (±0.01 m vertical uncertainty). Position control was provided by a handheld Garmin GPS unit (±3 m horizontal uncertainty). Data was cleaned and interpolated into a continuous bathymetric model using ArcGIS 10.0 revealing a simple bowl-like geometry with the deepest part of the hole located towards the center.

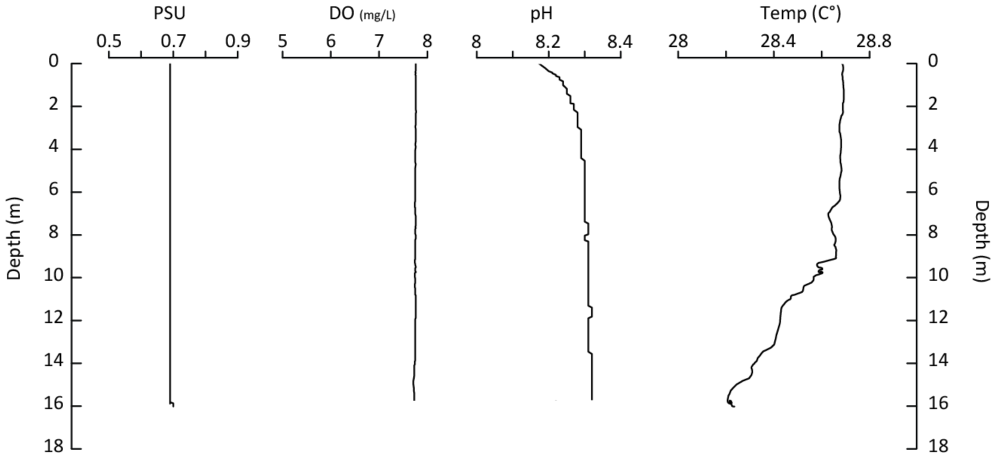

Figure S3. Hydrographic profiles from Cenote Muyil. From left to right: Salinity, dissolved oxygen, pH, and temperature. Water column structure was measured with a YSI EXO1 water quality sonde calibrated within 48 hours of deployment, which measures depth (±0.004 m), dissolved oxygen (±0.1 mg/L), pH (±0.1 pH units), temperature (±0.01°C), and salinity (±0.1 psu).

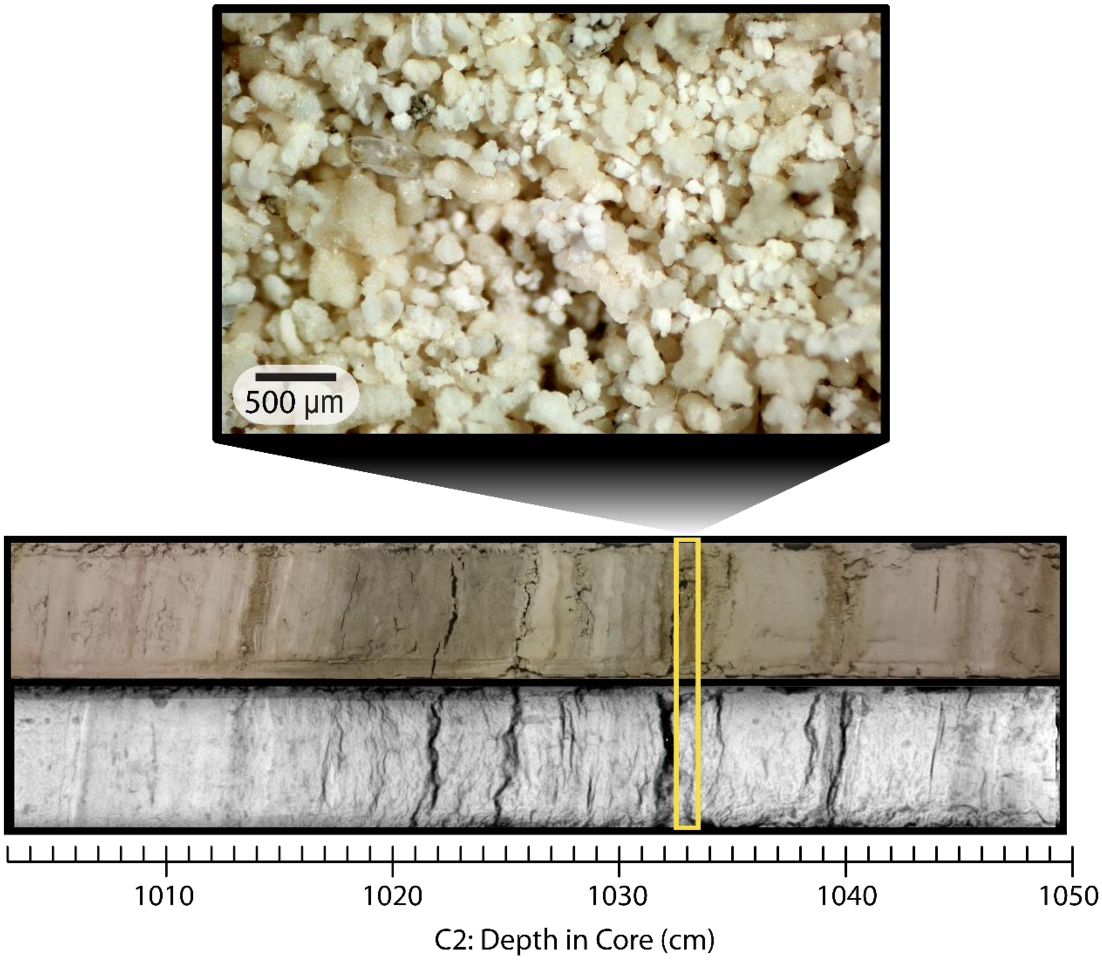

Figure S4. Example of amorphous coarse-grained (> 63µm diameter) carbonate sand particles and stratigraphy (X-radiograph and optical) from Muyil C2 (1003 cm down core). Yellow box highlights coarse bed shown in the callout.

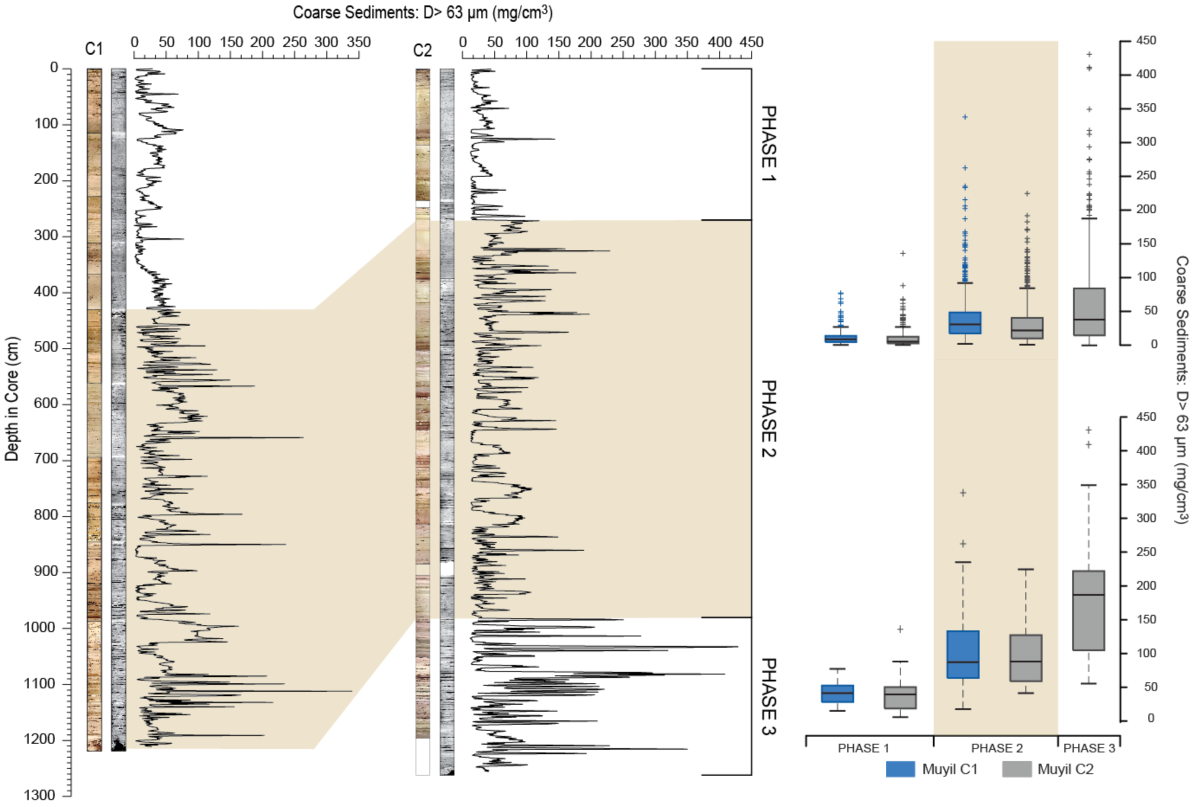

Figure S5. Left: Down core grain size plot showing three sedimentary phases. Top Right: Box and whisker plot showing grain size distribution for coarse beds exceeding 63µm. Boxes represent event deposit densities falling within the 25th and 75th percentiles. Internal lines denote median values. Whiskers encompass range of data not considered outliers. Crosses depict outliers. Bottom Right: Same as above except depicts only coarse beds that satisfy the criteria for event deposits.

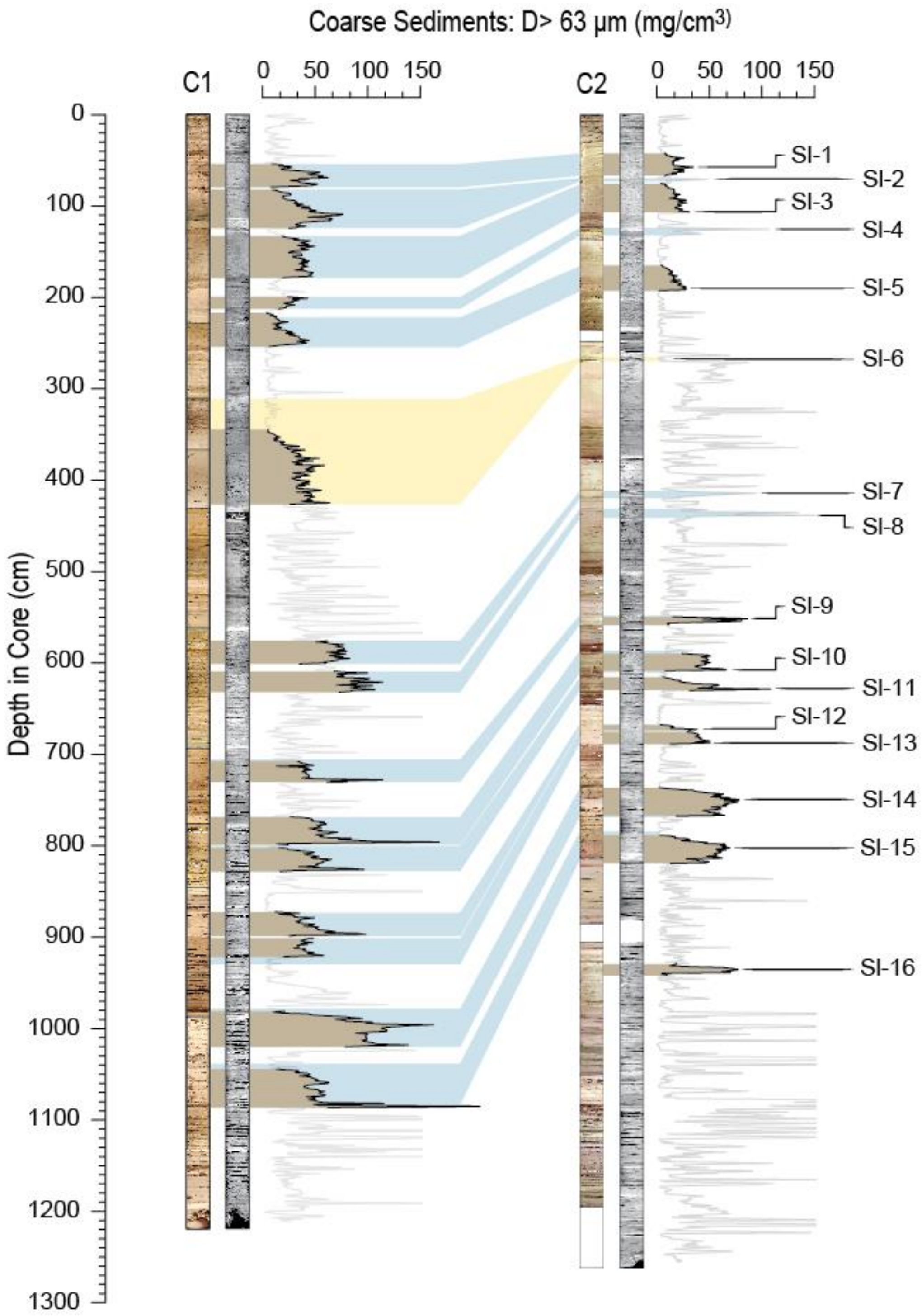

Figure S6. Numbered slump features between C1 (left) and C2 (right).

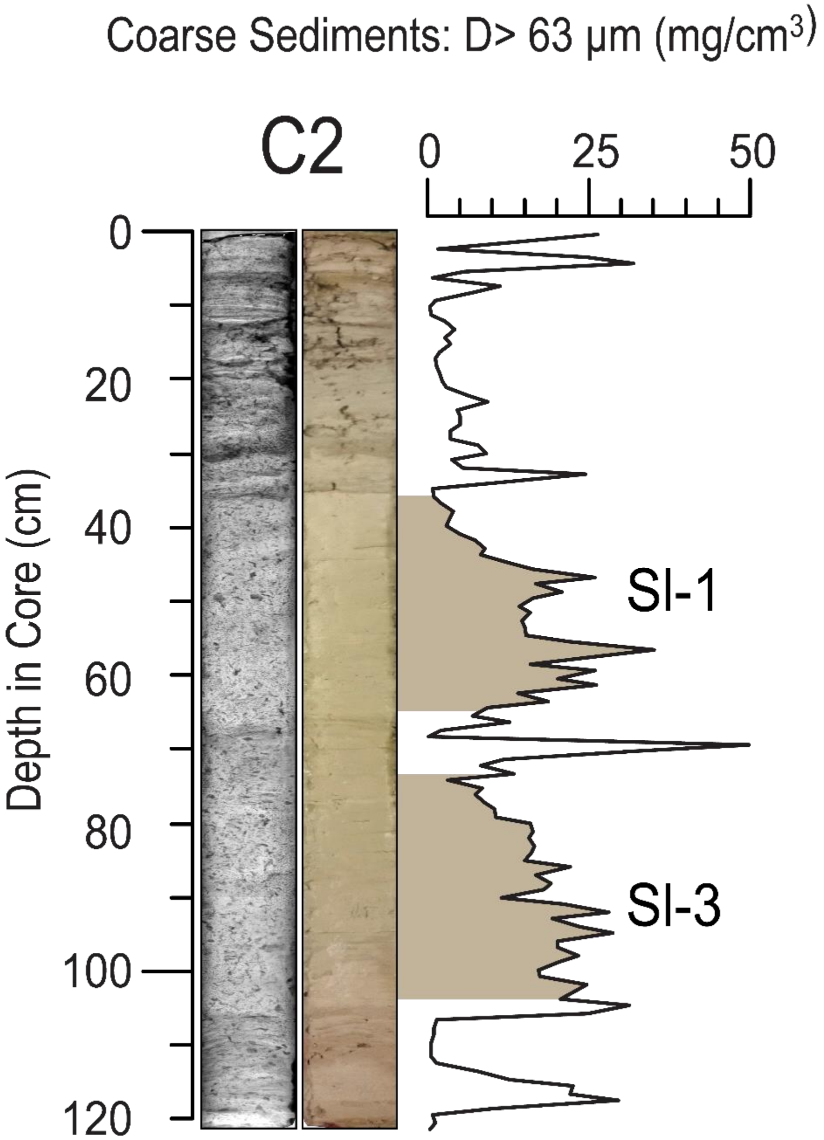

Figure S7. Upper 120 cm of core C2 (left: X-radiograph; right: optical image) showing ~homogenous Sl-1 and Sl-3 slump features separated by a slight change in sediment color likely related to slump Sl-2 (present in C1).

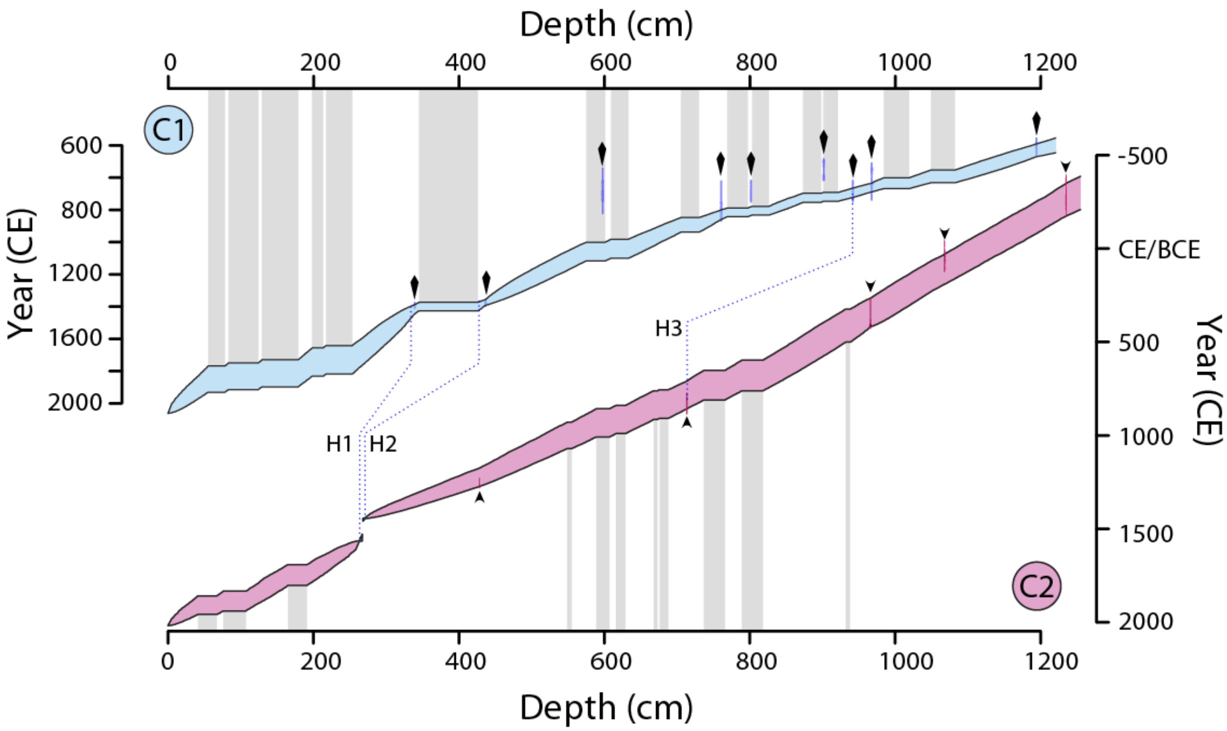

Figure S8. Age Models for C1 (cyan) and C2 (fuchsia). Grey bars denote instantaneous slump features. Diamonds (chevrons) depict depth of C1 (C2) dates. Age uncertainty for each date is indicated by narrow vertical bars. H1, H2, and H3 mark stratigraphic tie points.

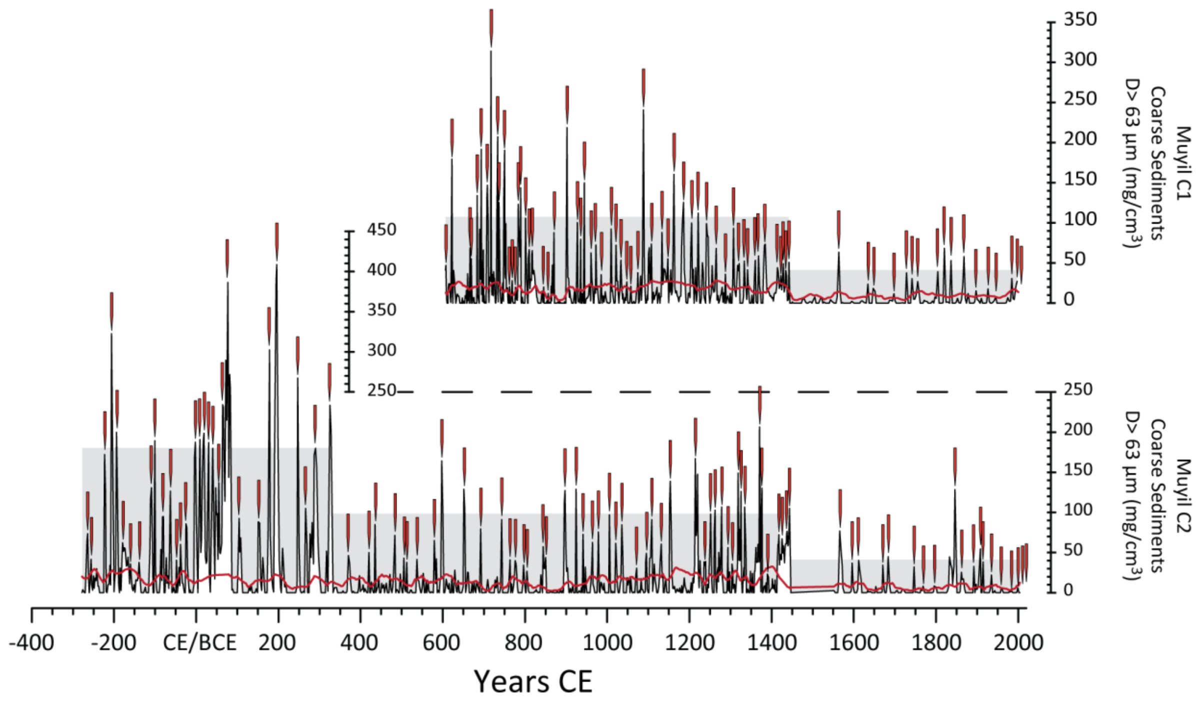

Figure S9. Coarse anomalies for C1 (top) and C2 (Bottom). Red line indicates smoothed background sedimentation subtracted from the raw coarse data to generate coarse-only signal (black). Red flags mark individual anomalous coarse deposits (C1: n = 74; C2: n = 99). Grey regions denote three sedimentary phases and mean event bed density for each phase.

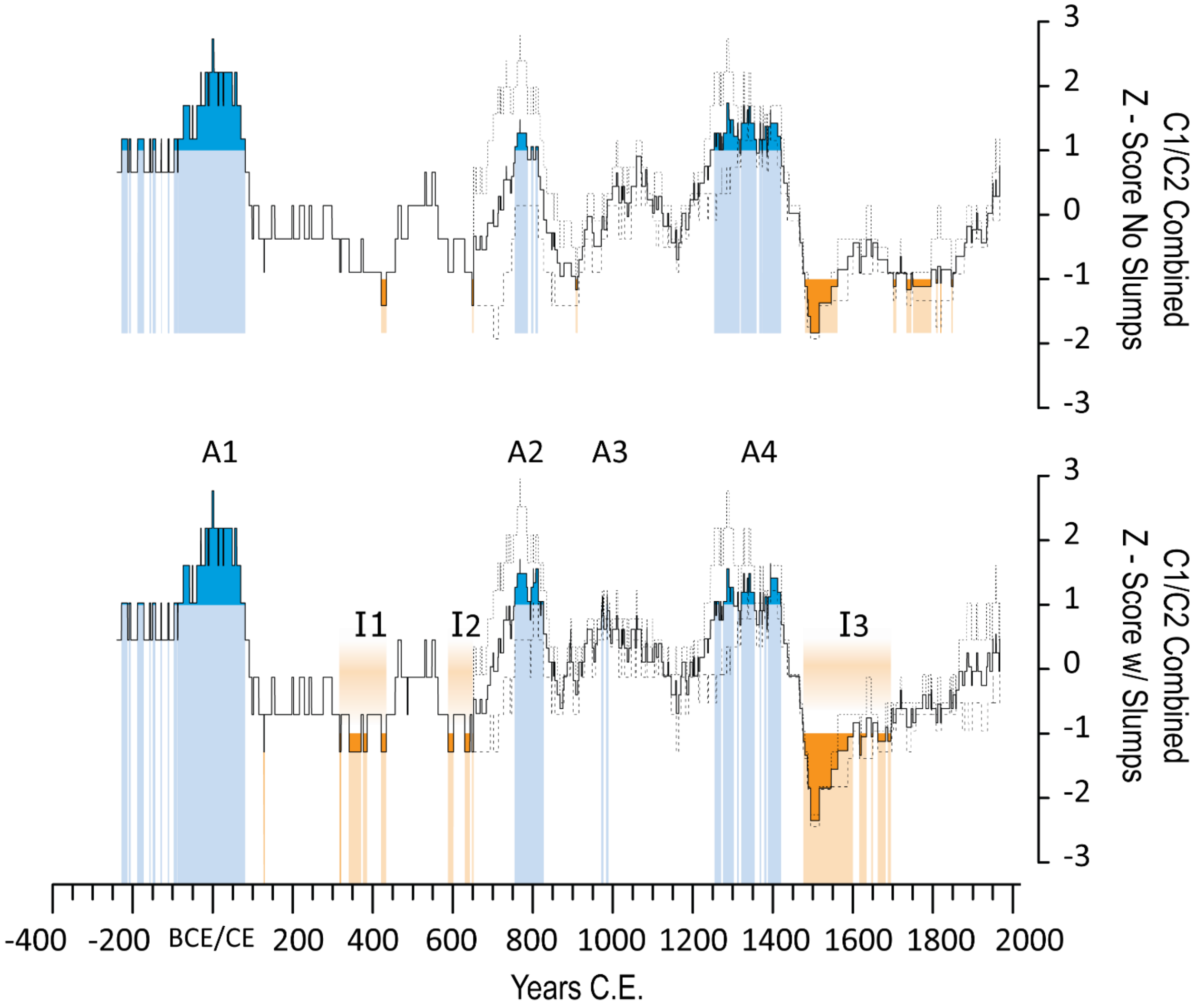

Figure S10. Top: Combined Z-Scores of C1 and C2 depositional event counts that exclude the instantaneous slump deposits. Bottom: Combined 100-year event count that considers slump deposits as indicative of storm events. All active intervals are depicted in both counts, with the exception of A3. The period between ~850 and 1000 CE records below, rather than above, average hurricane frequency when the slumps are omitted from the event count. While this disagreement does overlap with the Terminal Classic Phase (~800 to 1050 CE), both records show above average storminess at the onset and terminus of the TCP and start of the Postclassic period (~1000 CE).

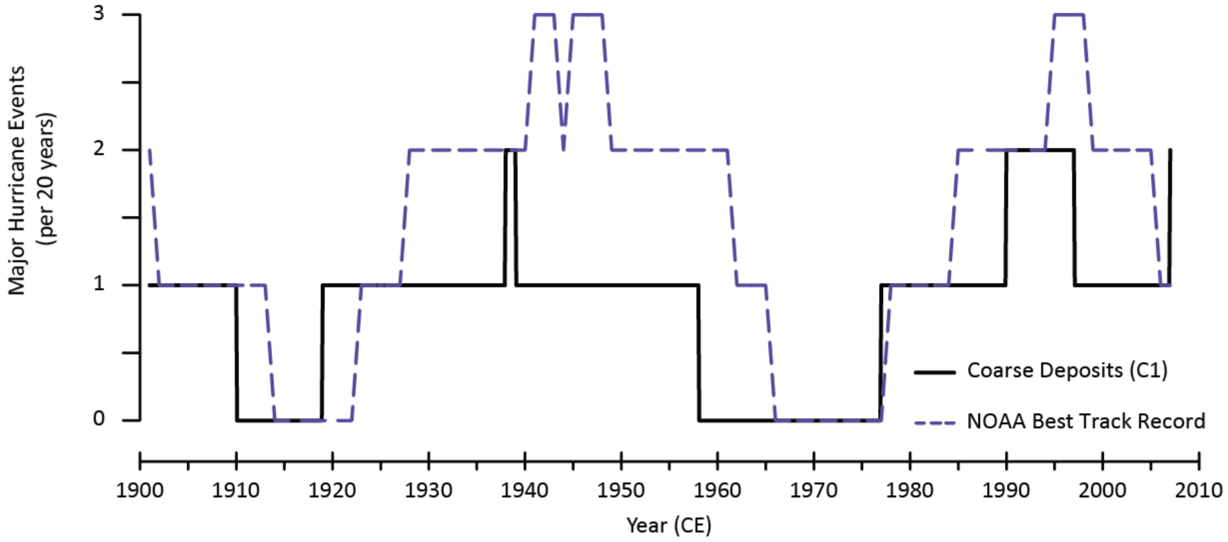

Figure S11. Multi-decadal (20-year) distribution of coarse deposits since 1900 CE from C1 compared with major events (≥ Cat 3 Saffir-Simpson Scale) passing within 100 km of Muyil as extracted from NOAA's Best Track historical hurricane database [36]. The positive correlation ($r = 0.58$, $p < 0.05$) indicates that coarse deposits in Cenote Muyil reflect proximal passing major storms. The correlation increases to $r = 0.7$ ($p < 0.05$) when we consider the most recent 60 years (1960 to present) of the historical archive when observational accuracy and monitoring methodologies improved.

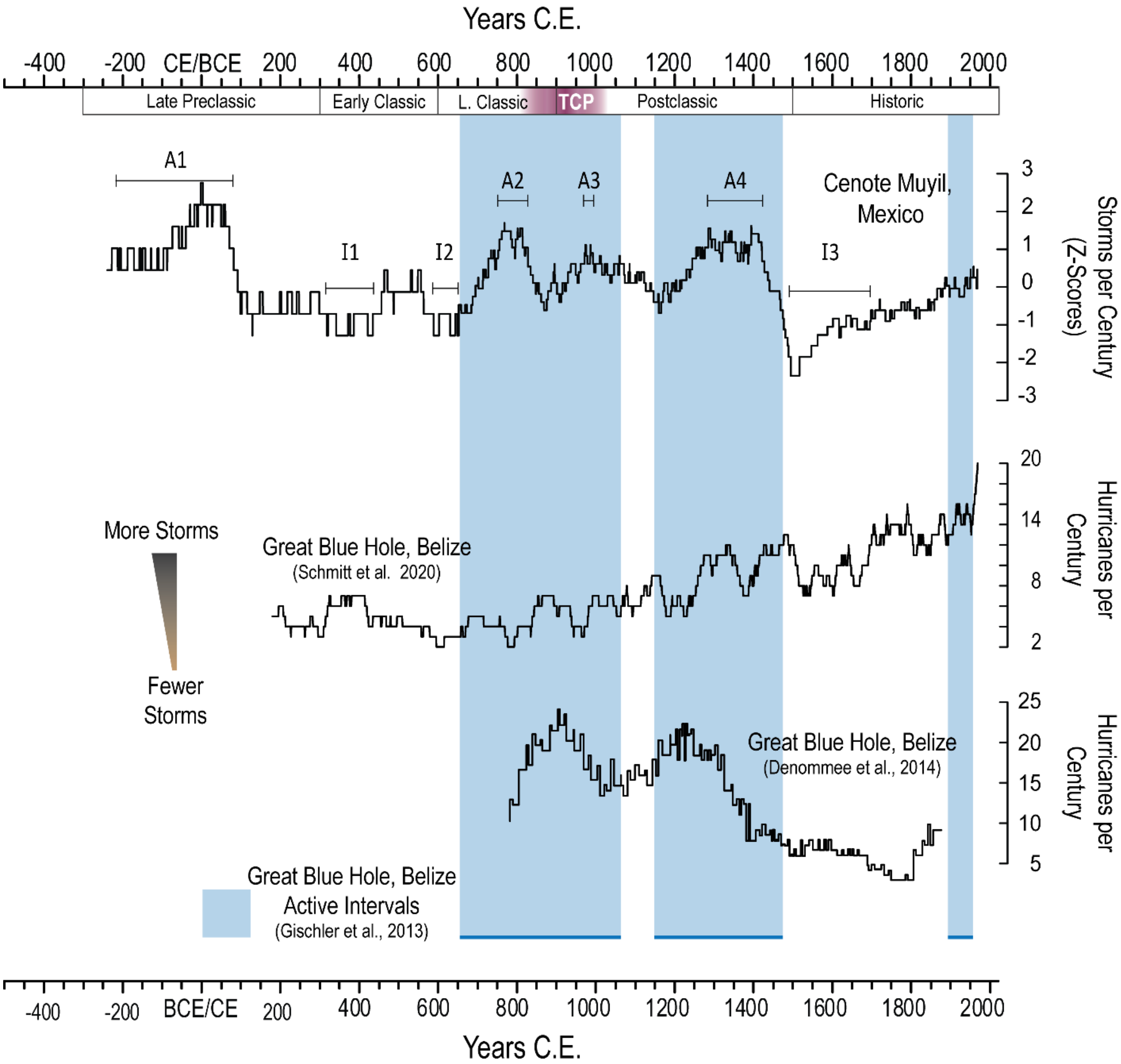

Figure S12. Muyil storm record (top) plotted against the three different reconstructions from Great Blue Hole, Belize. Schmitt, Gischler [37] (middle), Denommee, Bentley [38] (bottom), and Gischler, Anselmetti [39] (blue bars). The data for Schmitt et. al. [37] and Gischler et. al. [39] comes from the respective publications. Denommee et. al. [38] is adapted from Donnelly, Hawkes [40]. Muyil active intervals A2, A3, and A4 broadly correlate to the active intervals as defined by Gischler et al. (in blue), and in partial agreement with the Denommee et al. reconstruction. The Schmitt et al. record has limited agreement with other reconstructions and instead exhibits an increasing trend over the last millennium, including during the Little Ice Age, that may reflect processes unrelated to storminess (e.g., sea-level rise). The 2014 record shows that at ~900 CE the region experienced 25 hurricanes per century. However, the 2020 record shows that 900 CE averaged less than six hurricanes a century. The period of least activity depicted in the 2014 reconstruction occurs ~1800 CE with fewer than 5 storms per century recorded. This coincides with one of the most active intervals in the 2020 record with ~16 storms per century recorded.

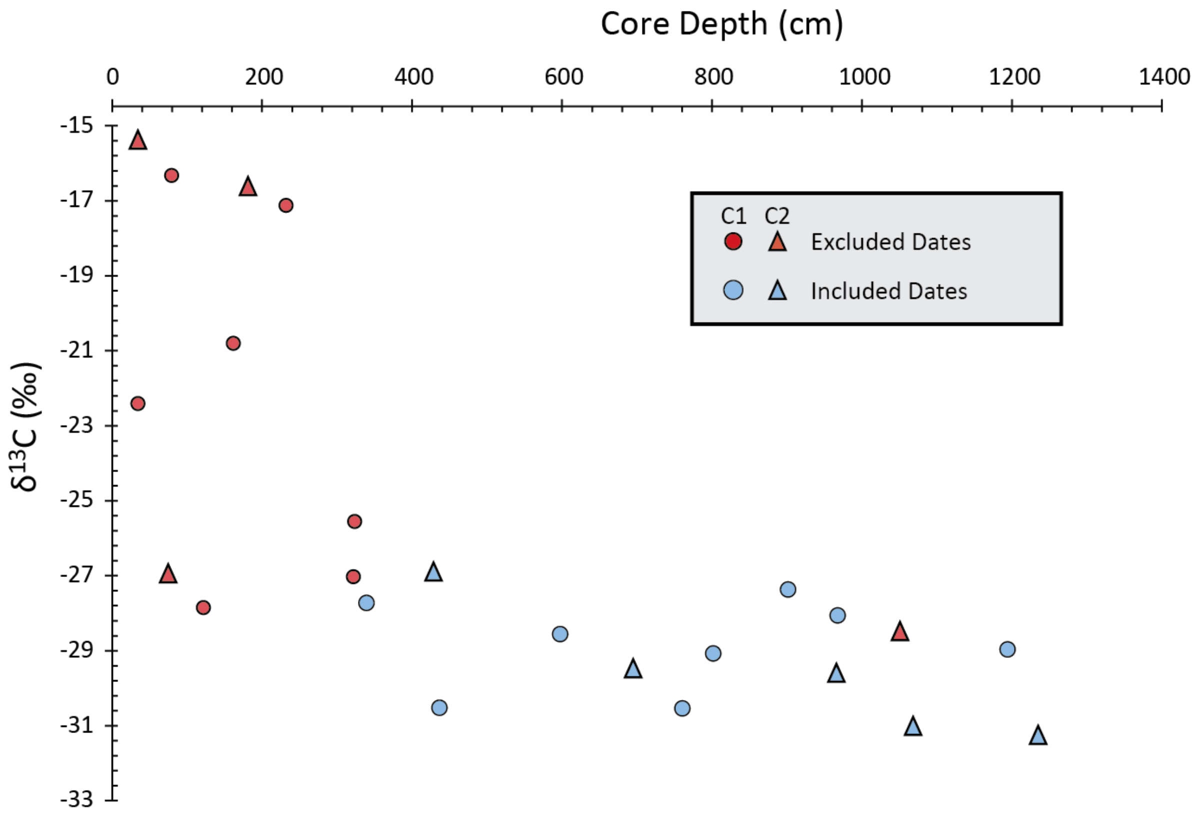

Figure S13. Measured D13C for each AMS date plotted against depth recovered in the core. Note that ten of the excluded dates fall within the upper 4 m of the cores. The six excluded dates within the top 2 m all have measured d13C values consistent with marine carbon and were ~2000 years older than would have been expected from the ages of the remaining AMS dates.

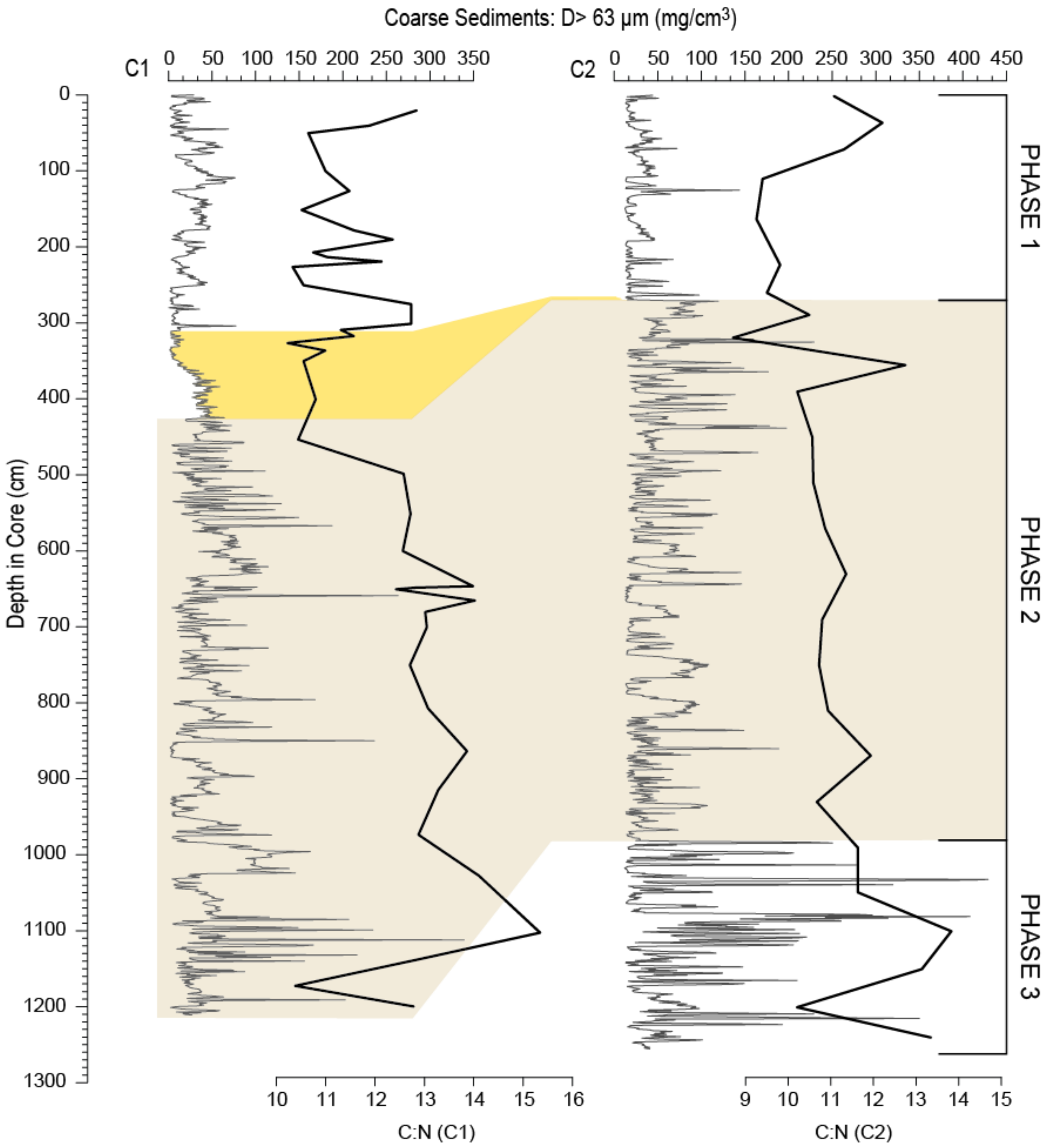

Figure S14. Three distinct sedimentary phases shown against the coarse grain and C:N variability. Slump Sl-6 highlighted in yellow. Reduced C:N values in the upper three meters are coincident with the Little Ice Age when fewer storms were available to transport terrestrial organic matter into the cenote for archival.

Supplementary Tables

Table S1. Mean sediment density (mg/cm^3) per phase for beds that satisfy the criteria for event deposits.

Phase	Muyil C1 (mean ± 1σ)	Muyil C2 (mean ± 1σ)
1	42.70 ± 18.5	42.19 ± 29.56
2	107.14 ± 63.81	98.27 ± 43.70
3		181.33 ± 98.26

Table S2. Mean C:N value for each of the three sedimentary phases. Phase 1 is divided into pre and post- 20th Century values to isolate the recent increase in C:N relative to the earlier parts of that interval.

Phase	Muyil C1 (C:N)	Muyil C2 (C:N)
1 (20^{thC})	12.84	11.53 ± 0.49
1	11.26 ± 0.76	9.49 ± 0.23
2	12.95 ± 1.09	10.82 ± 0.89
3		12.30 ± 1.25

Table S3. Radiocarbon dates used in this analysis (following page)

Cenote Muyil Radiocarbon Table

	Index No.	Accession number	Core	Core Section Depth (cm)	Total Core Depth (cm)	F14C	Conventional 14C age	d13C (‰)	1s calendar ages in yrs. B1950 (probability)	2s calendar ages in yrs. B1950 (probability)	Highest probability 1s age	Median probability age in yrs B_{1950}	Median probability age in Cal Year CE
**Muyil C1**	8	OS-152435	Muyil1-C1-4:12	28	339	0.9458 ± 0.0019	450 ± (15)	-27.72	512-501	520-495	505 ± 5	505	1445
	9	OS-151128	Muyil1-C1-6:12	5	436.5	0.941 ± 0.0019	490 ± (15)	-30.52	526-514	536-508	520 ± 5	520	1430
	10	OS-151129	Muyil1-C1-7:12	32.5	597.5	0.8621 ± 0.0022	1190 ± (20)	-28.55	1175-1174 (0.03)<br>1169-1162 (0.131)<br>1123-1069 (0.839)	1175-1158 (0.169)<br>1154-1061 (0.831)	1100 ± 25	1110	840
	11	OS-151130	Muyil1-C1-8:12	64.5	760.5	0.869 ± 0.0017	1130 ± (15)	-30.53	1059-1052 (0.148)<br>1031-992 (0.734)<br>983-977 (0.118)	1065-973 (0.997)<br>963-963 (0.003)	1010 ± 20	1010	940
	12	OS-151195	Muyil1-C1-9:12	21	801.5	0.8618 ± 0.0018	1200 ± (15)	-29.07	1175-1175 (0.019)<br>1166-1161 (0.102)<br>1127-1072 (0.879)	1175-1174 (0.02)<br>1169-1158 (0.118)<br>1154-1067 (0.862)	1100 ± 30	1110	840
	13	OS-151196	Muyil1-C1-10:12	52	901.5	0.8494 ± 0.0017	1310 ± (15)	-27.36	1283-1268 (0.405)<br>1206-1191 (0.442)<br>1184-1179 (0.153)	1288-1258 (0.415)<br>1256-1247 (0.065)<br>1209-1178 (0.52)	1200 ± 10	1210	740
	14	OS-151197	Muyil1-C1-10:12	118	967.5	0.8573 ± 0.0018	1240 ± (15)	-28.05	1243-1224 (0.4)<br>1218-1216 (0.039)<br>1176-1176 (0.03)<br>1159-1129 (0.532)	1264-1252 (0.032)<br>1248-1209 (0.425)<br>1177-1175 (0.03)<br>1163-1117 (0.452)<br>1097-1075 (0.061)	1140 ± 15	1160	790
	15	OS-151198	Muyil1-C1-12:12	55	1194	0.8303 ± 0.0018	1490 ± (15)	-28.96	1378-1351	1396-1345 (0.953)<br>1325-1315 (0.047)	1360 ± 15	1360	590
**Muyil C2**	19	OS-139797	Muyil2-C2-5:13	52.5	428.5	0.9102 ± 0.0019	755 ± (15)	-26.92	683-673	720-707 (0.068)<br>691-669 (0.932)	680 ± 5	680	1270
	20	OS-135560	Muyil2-C2-7:13	67.5	713.5	0.8608 ± 0.0017	1200 ± (15)	-28.01	1175-1175 (0.019)<br>1166-1161 (0.102)<br>1127-1072 (0.879)	1175-1174 (0.02)<br>1169-1158 (0.118)<br>1154-1067 (0.862)	1100 ± 30	1110	840
	21	OS-135561	Muyil2-C2-11:13	60	966	0.8129 ± 0.0017	1660 ± (15)	-29.65	1567-1564 (0.062)<br>1557-1557 (0.016)<br>1547-1530 (0.922)	1684-1679 (0.016)<br>1587-1522 (0.984)	1540 ± 10	1540	410
	23	OS-152332	Muyil2-C2-12:13	18	1068	0.7828 ± 0.0022	1970 ± (25)	-31.04	1932-1869 (0.877)<br>1851-1841 (0.123)	1985-1963 (0.093)<br>1946-1860 (0.733)<br>1858-1830 (0.175)	1900 ± 30	1900	50
	24	Beta - 505291	Muyil2-C2-13:13	53	1235	0.7661 ± 0.0029	2240 ± (30)	-31.3	2329-2301 (0.294)<br>2236-2177 (0.625)<br>2167-2159 (0.08)	2338-2294 (0.273)<br>2268-2152 (0.727)	2210 ± 30	2230	-280
**Ommitted Inconsistent Dates**	1	OS-153828	Muyil1-C1-1:12	34.5	34.5	0.7871 ± 0.0017	1920 ± (20)	-15.43	1881-1821 (0.896)<br>1805-1797 (0.074)<br>1755-1752 (0.031)	1918-1913 (0.013)<br>1889-1745 (0.987)	1850 ± 30	1840	110
	2	OS-152360	Muyil1-C1-1:12	34.5	34.5	0.9027 ± 0.0036	820 ± (30)	-22.4	732-687	782-781 (0.006)<br>775-755 (0.109)<br>751-678 (0.886)	710 ± 25	715	1235
	3	OS-153829	Muyil1-C1-2:12	5	121.5	0.9611 ± 0.0019	320 ± (15)	-27.85	429-421 (0.133)<br>411-369 (0.636)<br>365-364 (0.025)<br>328-315 (0.205)	447-352 (0.804)<br>333-310 (0.196)	390 ± 20	385	1565
	4	OS-152361	Muyil1-C1-2:12	45.5	161.5	0.7569 ± 0.003	2240 ± (30)	-20.8	2329-2301 (0.294)<br>2236-2177 (0.625)<br>2167-2159 (0.08)	2338-2294 (0.273)<br>2268-2152 (0.727)	2210 ± 30	2230	-280
	5	OS-151126	Muyil1-C1-3:12	4	232	0.7695 ± 0.0017	2110 ± (15)	-17.12	2110-2050 (0.891)<br>2016-2009 (0.109)	2122-2036 (0.793)<br>2033-2033 (0.003)<br>2031-2002 (0.204)	2080 ± 30	2070	-120
	6	OS-152362	Muyil1-C1-4:12	10.5	321.5	0.9335 ± 0.0025	555 ± (20)	-27.02	622-613 (0.23)<br>554-532 (0.77)	625-596 (0.378)<br>557-528 (0.622)	545 ± 10	550	1400
	7	OS-151127	Muyil1-C1-4:12	12.5	323.5	0.7325 ± 0.0016	2500 ± (20)	-25.55	2709-2696 (0.122)<br>2637-2625 (0.111)<br>2623-2614 (0.086)<br>2592-2515 (0.68)	2721-2673 (0.199)<br>2655-2652 (0.007)<br>2646-2612 (0.183)<br>2600-2494 (0.61)	2550 ± 40	2580	-630
	16	Beta - 505290	Muyil2-C2-1:13	74.5	74.5	0.9315 ± 0.0035	600 ± (30)	-27	637-590 (0.826)<br>562-553 (0.174)	649-581 (0.745)<br>572-543 (0.255)	615 ± 25	600	1350
	17	OS-137781	Muyil2-C2-1:13	79	79	0.7559 ± 0.0016	2250 ± (15)	-16.32	2330-2306 (0.43)<br>2228-2206 (0.346)<br>2195-2182 (0.223)	2337-2300 (0.374)<br>2243-2243 (0.002)<br>2240-2157 (0.624)	2320 ± 10	2220	-270
	18	OS-137782	Muyil2-C2-3:13	48	181	0.7475 ± 0.0017	2340 ± (20)	-16.67	2353-2343	2399-2397 (0.005)<br>2363-2332 (0.995)	2350 ± 5	2350	-400
	22	OS-152331	Muyil2-C2-12:13	0.5	1050.5	0.7303 ± 0.002	2530 ± (20)	-28.54	2726-2701 (0.416)<br>2631-2617 (0.216)<br>2582-2571 (0.11)<br>2561-2541 (0.258)	2736-2697 (0.344)<br>2638-2615 (0.187)<br>2592-2511 (0.451)<br>2508-2508 (0.002)<br>2506-2499 (0.016)	2710 ± 15	2620	-670

Supplemental References

1. Tamalavage, A.E., et al., *Organic matter sources and lateral sedimentation in a Bahamian karst basin (sinkhole) over the late Holocene: Influence of local vegetation and climate.* Palaeogeography, Palaeoclimatology, Palaeoecology, 2018. **506**: p. 70-83.
2. Brown, A.L., et al., *A coastal Yucatan sinkhole records intense hurricane events.* Journal of Coastal Research, 2014. **30**(2): p. 418-428.
3. van Hengstum, P.J., et al., *Holocene sedimentation in a blue hole surrounded by carbonate tidal flats in The Bahamas: Autogenic versus allogenic processes.* Marine geology, 2020. **419**: p. 106051.
4. van Hengstum, P.J., et al., *The intertropical convergence zone modulates intense hurricane strikes on the western North Atlantic margin.* Scientific Reports, 2016. **6**: p. 21728.
5. van Hengstum, P.J., et al., *Drought in the northern Bahamas from 3300 to 2500 years ago.* Quaternary Science Reviews, 2018. **186**: p. 169-185.
6. Lamb, A.L., G.P. Wilson, and M.J. Leng, *A review of coastal palaeoclimate and relative sea-level reconstructions using δ13C and C/N ratios in organic material.* Earth-Science Reviews, 2006. **75**(1-4): p. 29-57.
7. Reimer, P.J., et al., *The IntCal20 Northern Hemisphere radiocarbon age calibration curve (0–55 cal kBP).* Radiocarbon, 2020. **62**(4): p. 725-757.
8. Blaauw, M. and J.A. Christen, *Flexible paleoclimate age-depth models using an autoregressive gamma process.* Bayesian Analysis, 2011. **6**(3): p. 457-474.
9. O'Leary, M.H., *Carbon isotopes in photosynthesis.* Bioscience, 1988. **38**(5): p. 328-336.
10. Gonneea, M.E., A. Paytan, and J.A. Herrera-Silveira, *Tracing organic matter sources and carbon burial in mangrove sediments over the past 160 years.* Estuarine, Coastal and Shelf Science, 2004. **61**(2): p. 211-227.
11. Wallace, E.J., et al. *Sedimentary Reconstructions of Tropical Cyclone Activity over the Past 1500 Years from Blue Holes in the Caribbean*. in *AGU Fall Meeting Abstracts*. 2017.
12. Winkler, T.S., et al., *Revising evidence of hurricane strikes on Abaco Island (The Bahamas) over the last 700 years.* Scientific Reports, 2020. **10**(1): p. 1-17.
13. Kenter, J.A.M., *Carbonate platform flanks: slope angle and sediment fabric.* Sedimentology, 1990. **37**(5): p. 777-794.
14. Horton, B.P., V. Rossi, and A.D. Hawkes, *The sedimentary record of the 2005 hurricane season from the Mississippi and Alabama coastlines.* Quaternary International, 2009. **195**(1-2): p. 15-30.
15. Hawkes, A.D., et al., *Sediments deposited by the 2004 Indian Ocean tsunami along the Malaysia–Thailand Peninsula.* Marine Geology, 2007. **242**(1-3): p. 169-190.
16. James, R.T., et al., *Hurricane effects on a shallow lake ecosystem, Lake Okeechobee, Florida (USA).* Fundamental and Applied Limnology, 2008. **172**(4): p. 273-287.
17. Ponte, A.L., *Periodic wind-driven circulation in an elongated and rotating basin.* Journal of Physical Oceanography, 2010. **40**(9): p. 2043-2058.
18. Ji, Z.G. and K.R. Jin, *Gyres and seiches in a large and shallow lake.* Journal of Great Lakes Research, 2006. **32**(4): p. 764-775.

19. Chimney, M.J., *Surface seiche and wind set-up on Lake Okeechobee (Florida, USA) during Hurricanes Frances and Jeanne.* Lake and Reservoir Management, 2005. **21**(4): p. 465-473.
20. Kelly, D.M., et al., *Validation of the FAST forecast model for the storm surges due to hurricanes Wilma and Ike.* Natural Hazards, 2016. **83**(1): p. 53-74.
21. Carter, D., *Prediction of wave height and period for a constant wind velocity using the JONSWAP results.* Ocean Engineering, 1982. **9**(1): p. 17-33.
22. Lagomasino, D., et al., *Connecting groundwater and surface water sources in groundwater dependent coastal wetlands and estuaries: Sian Ka'an Biosphere Reserve, Quintana Roo, Mexico.* Estuaries and Coasts, 2015. **38**(5): p. 1744-1763.
23. Aragón-Moreno, A.A., et al., *Middle and late Holocene mangrove dynamics of the Yucatan Peninsula, Mexico.* Journal of South American Earth Sciences, 2018. **85**: p. 307-311.
24. Curtis, J.H., D.A. Hodell, and M. Brenner, *Climate variability on the Yucatan Peninsula (Mexico) during the past 3500 years, and implications for Maya cultural evolution.* Quaternary Research, 1996. **46**(1): p. 37-47.
25. Chaudhuri, P., S. Chaudhuri, and R. Ghosh, *The role of mangroves in coastal and estuarine sedimentary accretion in Southeast Asia.* Sedimentary Processes-Examples from Asia, Turkey and Nigeria, 2019.
26. Furukawa, K. and E. Wolanski, *Sedimentation in mangrove forests.* Mangroves and Salt Marshes, 1996. **1**(1): p. 3-10.
27. Wolanski, E., *Hydrodynamics of mangrove swamps and their coastal waters.* Hydrobiologia, 1992. **247**(1): p. 141-161.
28. Scoffin, T.P., *The trapping and binding of subtidal carbonate sediments by marine vegetation in Bimini Lagoon, Bahamas.* Journal of Sedimentary Research, 1970. **40**(1).
29. Jaijel, R., et al., *Shallow geophysical exploration at the ancient maritime Maya site of Vista Alegre, Yucatan Mexico.* Journal of Archaeological Science: Reports, 2018. **19**: p. 52-63.
30. Jaijel, R., et al., *Coastal reconstruction of Vista Alegre, an ancient maritime Maya settlement.* Palaeogeography, Palaeoclimatology, Palaeoecology, 2018. **497**: p. 25-36.
31. Krywy-Janzen, A., et al., *Water-level change recorded in Lake Pac Chen Quintana Roo, Mexico infers connection with the aquifer and response to Holocene sea-level rise and Classic Maya droughts.* Journal of Paleolimnology, 2019. **62**(4): p. 373-388.
32. Coutino, A., et al., *Inland tidal oscillations within the Yucatan Peninsula.* Geophysical Research Letters, 2021. **48**(9): p. e2020GL092091.
33. Khan, N.S., et al., *Drivers of Holocene sea-level change in the Caribbean.* Quaternary Science Reviews, 2017. **155**: p. 13-36.
34. Witschey, W.R.T., *The archaeology of Muyil, Quintana Roo, Mexico: a Maya site on the east coast of the Yucatan peninsula*. 1993, Tulane University.
35. Schneider, U., et al., *GPCC full data reanalysis version 6.0 at 0.5: Monthly land-surface precipitation from rain-gauges built on GTS-based and historic data.* GPCC Data Rep., doi, 2011. **10**.
36. Knapp, K.R., et al., *The international best track archive for climate stewardship (IBTrACS) unifying tropical cyclone data.* Bulletin of the American Meteorological Society, 2010. **91**(3): p. 363-376.

37. Schmitt, D., et al., *Caribbean cyclone activity: an annually-resolved Common Era record.* Scientific Reports, 2020. **10**(1): p. 1-17.
38. Denommee, K., S. Bentley, and A. Droxler, *Climatic controls on hurricane patterns: a 1200-y near-annual record from Lighthouse Reef, Belize.* Scientific Reports, 2014. **4**: p. 3876.
39. Gischler, E., F.S. Anselmetti, and E.A. Shinn, *Seismic stratigraphy of the Blue Hole (Lighthouse Reef, Belize), a late Holocene climate and storm archive.* Marine Geology, 2013. **344**: p. 155-162.
40. Donnelly, J.P., et al., *Climate forcing of unprecedented intense-hurricane activity in the last 2000 years.* Earth's Future, 2015. **3**(2): p. 49-65.
